# A Study on the Dilational Modulus Measurement of Polyacrylic Acid Films at Air–Water Interface by Pendant Bubble Tensiometry

**DOI:** 10.3390/polym16101359

**Published:** 2024-05-10

**Authors:** Johann Eduardo Maradiaga Rivas, Li-Jen Chen, Shi-Yow Lin, Siam Hussain

**Affiliations:** 1 Department of Chemical Engineering, National Taiwan University of Science and Technology, 43, Sec. 4, Keelung Road, Taipei 106, Taiwan; m11006829@mail.ntust.edu.tw; 2 Department of Chemical Engineering, National Taiwan University, 1, Sec. 4, Roosevelt Road, Taipei City 106, Taiwan; ljchen@ntu.edu.tw

**Keywords:** dilational modulus, polymer film, surface tension, pendant bubble tensiometry, natural perturbation, polyacrylic acid

## Abstract

The dilational modulus (E) of polymer films has been commonly measured using the oscillating ring/bubble/drop methods with an external force, and often without specifying the state of the adsorbed film. This study explores an approach where E was determined from the relaxations of surface tension (ST) and surface area (SA) of natural perturbations, in which ST and SA were monitored using a pendant bubble tensiometer. The E of the adsorbed film of PAA (polyacrylic acid) was evaluated for aqueous solutions at C_PAA_ = 5 × 10^−4^ g/cm^3^, [MW = 5, 25, and 250 (kDa)]. The E (=dγ/dlnA) was estimated from the surface dilational rate (dlnA/dt) and the rate of ST change (dγ/dt) of the bubble surface from the natural perturbation caused by minute variations in ambient temperature. The data revealed that (i) a considerable time is required to reach the equilibrium-ST (γ_eq_) and to attain the saturated dilational modulus (E_sat_) of the adsorbed PAA film, (ii) both γ_eq_ and E_sat_ of PAA solutions increase with MW of PAA, (iii) a lower MW solution requires a longer time to reach its γ_eq_ and E_sat_, and (iv) this approach is workable for evaluating the E of adsorbed polymer films.

## 1. Introduction

In recent years, there has been growing attention to water-soluble, super-absorbent polymers (SAPs) such as polyvinyl alcohol (PVA), polyethylene glycol (PEG), and polyacrylic acid (PAA), due to their unique properties, including surface activity, high water solubility, biocompatibility, and biodegradability [1]. Amongst these SAPs, PAA stands out as one of the most widely utilized due to its diverse applications in various industrial and scientific sectors. SAPs find extensive use in colloid stabilization [1,2], controlled drug delivery [2,3,4], water treatment [5,6], and environmental remediation [7,8,9]. PAA, in particular, exhibits remarkable versatility owing to its inherent properties, including its ability to acquire a net negative charge in solution through the presence of carboxyl groups. This versatility, coupled with its high molecular weight, chain flexibility, and solubility in aqueous media, makes PAA a cornerstone in the realm of anionic polymers, extensively employed in dispersion compounds and SAP formulations [10,11].

PAA’s significance extends beyond its conventional applications, as its polyelectrolyte behavior further amplifies its utility in diverse contexts. The presence of carboxyl groups within the PAA polymer chain enables it to exhibit weak polyelectrolyte characteristics, with the ability to undergo partial dissociation in solution, resulting in the formation of negatively charged carboxylate ions. This ionization behavior not only influences the electrostatic interactions and intermolecular associations of PAA but also plays a pivotal role in its surface behavior [12,13,14].

Furthermore, PAA’s polyelectrolyte behavior holds significant real-life relevance in a multitude of fields. Studies have elucidated how the extent of ionization of PAA influences its interactions with other species, such as ions, surfactants, and biological molecules, thereby impacting processes like drug delivery [15,16,17], colloid stabilization [18,19], and water treatment [20]. For instance, the ability of PAA to form complexes with metal ions or oppositely charged polymers has been exploited for the removal of heavy metals from water sources [21]. Additionally, the ionization state of PAA, owing to its weak polyelectrolytic behavior, could likely impact its adsorption onto interfaces; thereby influencing its dilational and shear rheological properties. 

The dilational modulus (E) of adsorbed polymeric films (like that of PAA) is influenced by the polymer structure [22,23,24] and solution composition (e.g., concentration, surface pressure, MW, additive, and pH) [25,26,27,28,29,30,31,32,33,34,35,36]. E was found to increase with increasing (i) surface pressure [25,26,27], (ii) polymer MW [26,27], (iii) salt concentration [31], and (iv) solution pH [35]. In addition, it was also reported [22,27,28,29,30,31,32,33,34] that with an increasing concentration of polymer or surfactant, E initially decreases at low concentrations (10^−5^–10^−2^ wt%), and then increases at higher concentrations (10^−2^–10^−1^ wt%). These dependencies are summarized in Table 1 and Table S1 in the Supplementary Materials. 

Most studies evaluate E from interfacial perturbations induced by an external force. Bykov et al. [23,28] utilized the oscillating ring method to examine the ST and E of solutions containing PMA/PAA + DTAB/C_n_TAB. Aricov et al. [24] employed the oscillating bubble method to monitor the ST and E of PAA (grafted with decyl and dodecyl chains) solutions. Okumura and Kawaguchi [25] applied the oscillating barrier and Wilhelmy plate to evaluate the E of PNIPAM solutions. However, no study considered the potential effects of such external force-induced perturbations on the E measurement. 

Moreover, the above reports generally did not specify the state of the adsorbed polymer films when E was evaluated, i.e., whether the solution had reached its equilibrium state, or whether the ST had reached its equilibrium value (γ_eq_), even if some of the lifetimes of polymer films (t_life_) were specified. The t_life_ at which E was measured was either 5–8 h [23,28,30,31,32,33], 15–20 h [24,29,35], or remained undisclosed [25,26,27]; but specifics on the state of the film are unclear. Diez-Pascual et al. [27] evaluated the E of PPG-r-PEG films as a function of frequency and surface pressure, though the state of the adsorbed film was not stated at all. It is crucial to note that a different state of the adsorbed film might correspond to a different E value. This lack of state information may introduce a large uncertainty on the reported E.

Given the above concerns, this study evaluated the dilational modulus of PAA films by monitoring the variations of ST and SA from a natural perturbance driven by minute variations in ambient temperature using a pendant bubble tensiometer. For each distinct perturbance, E was calculated from the surface dilational rate (dlnA/dt) and the rate of ST change (dγ/dt) of the bubble surface (E_i_ = dγ/dlnA). The variation in E_i_ was then examined at numerous t_life_ (i.e., at various states of the film) throughout the PAA adsorption: from a clean air–water interface, through γ_eq_, and beyond. The data revealed that it takes a very long time for the E of the adsorbed polymer film to reach its saturated state (E_sat_). Moreover, both E_sat_ and γ_eq_ increase with PAA MW.

## 2. Materials and Methods

**Material and Solution.** Polyacrylic acid (powder, CAS number 9003-01-4, molecular weights of 5, 25, and 250 kDa, and product numbers 192031, 181293, and 181285, respectively) was purchased from Sigma-Aldrich, St. Louis, MO, USA and used as received. Pure water (with a specific conductance of κ < 0.057 μS/cm, obtained from the UP-DQ Plus System from Pure Yes Ltd., Taipei, Taiwan) was used to prepare the PAA_(aq)_ solutions with different MW at a fixed wt% concentration, C_PAA_ = 5 × 10^−4^ g/cm^3^. PAA_(aq)_ solution, 28 mm^3^, was poured into the quartz cell (22 × 42 × 44 mm^3^) for ST measurement. 

**Tensiometer.** The relaxations of surface tension (ST) and surface area (SA) of an air bubble inside the PAA solutions at 25 °C were monitored by using a video-enhanced pendant bubble tensiometer. The equipment and operation procedure are detailed in Section S2 of the Supplementary Materials, an illustration in Figure S1, and references [37,38]. When the PAA solution was introduced into the quartz cell and positioned on an adjustable stage, it was allowed to stand still for ~10 min to reach a static state. A pendant air bubble with a diameter of ~2 mm was formed at the center of the solution in ~2 s with an inverted stainless-steel needle (18-gauge, O.D = 1.27 mm, I.D = 0.84 mm). Sequential images of the bubble were taken and then processed to determine the edge coordinates, which were then fitted with the Young–Laplace equation to determine the ST and bubble SA.

**Surface perturbation.** The quartz cell and PAA solution were placed within a thermostatic chamber. During the entire ST measurement, a closed system (the air within the pendant bubble and the Teflon tubing section connecting the valve to the needle) was established. The external temperature was maintained at a steady 25 ± 1 °C. Meanwhile, the temperature within the chamber (T_s_ and T_air_) experienced fluctuations over time, which could have led to minor changes in the bubble’s volume and SA. Note that (i) the repeatability of the ST measurement was ~0.1 mN/m [39], and (ii) during the later stage of the adsorption process, a forced perturbation (either a rapid compression or expansion of the pendant bubble) was conducted to confirm if the ST returned to its previous value.

**Figure 1:**
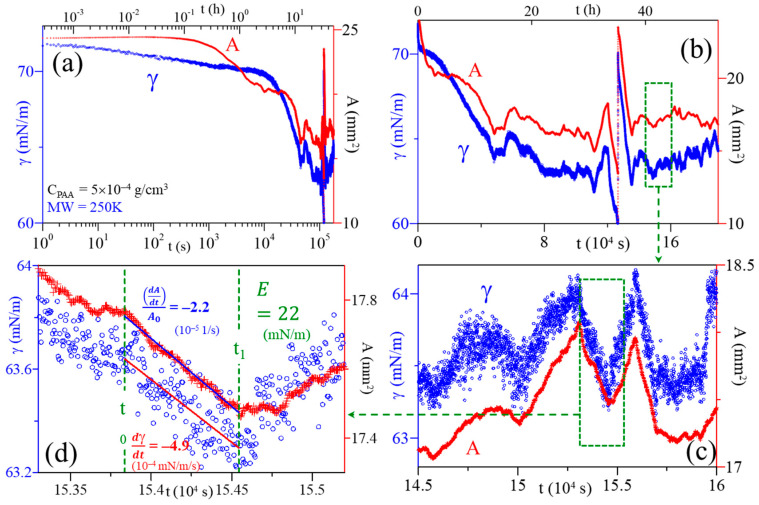
(**a**,**b**) Relaxations of ST (γ) and SA (A) of a pendant bubble for a purely aqueous PAA solution at C_PAA_ = 5 × 10^–4^ g/cm^3^ (MW 250 kDa). (**c**) Relaxations of ST and SA at t = 14.5–16.0 (10^4^ s), depicting the fluctuations in SA and ST. (**d**) Illustration of the fitting to evaluate E_i_ for an adsorbed polymer film.

**Dilational modulus.** The dilational modulus was evaluated following the manner in ref. [40]. After obtaining the complete SA and ST relaxations for a PAA solution (as illustrated in Figure 1a,b for PAA solution of MW 250 kDa; C = 5 × 10^−4^ g/cm^3^), the fitting process was performed by identifying marked fluctuations in SA and ST, which resulted from the changes in ambient temperature. The surface dilational modulus was estimated from the variation in ST and SA relaxation, as illustrated in Figure 1c,d, at t = 15.384–15.454 (10^4^ s), using the following steps:

1. The individual perturbances are identified in regions where SA and ST relaxations both have nearly linear changes (∆A > ~0.05 mm^2^) and ST (∆γ > ~0.1 mN/m);
2. The onset and end of the perturbance are carefully selected (t_0_ and t_1_), which in turn will correspond to specific A_0_, A_1_, γ_0_, γ_1_ values, respectively;
3. A linear regression is applied to the SA and ST data, respectively, in the selected time interval. The slopes from each linear fit provide the values for dγ/dt and dA/dt; and
4. The surface dilational rate (dlnA/dt) is then obtained by evaluating dlnA/dt = (dA/dt)/(A_0_) and the dilational modulus (E_i_) for an individual perturbance is calculated from E_i_ = (dγ/dt)/(dlnA/dt).

This fitting process is applied throughout the entire SA and ST relaxation of each polymer solution studied and many E_i_ can be obtained for each ST perturbance. Three more examples are given in Figure S2 for C = 5 × 10^−4^ g/cm^3^ (MW 25 kDa) at t = 8.197–8.247 (p1), 27.83–28.10 (p2), and 29.10–29.15 (p3) (10^4^ s). 

An average dilational modulus (E_avg_) is evaluated to show the state of the interface over a considerably longer time. E_avg_ over the interval of several individual perturbances (listed in Table 2 and Table S2, and Figure S3c) was calculated from the slope of the best fitting line in the plot between dγ/dt (rate of surface tension change) and dlnA/dt (relative surface expansion rate), as illustrated in Figure S2d at t = 20.47–21.35 (10^4^ s) for a solution of MW 5 kDa. Two additional examples of the evaluation of E_avg_ were given in Figures S4 and S5 for MW 25 and 250 kDa.

## 3. Results

A video-enhanced pendant bubble tensiometer was used to monitor the dynamic/equilibrium ST of PAA_(aq)_ solutions at C = 5 × 10^−4^ g/cm^3^ (MW 5, 25, and 250 kDa) at 25 °C. The measurement began from a clean air–water interface and continued through equilibrium-ST (γ_eq_), and up to ~20 h beyond γ_eq_. Small perturbances were identified and analyzed to evaluate the dilational modulus, E = (dγ/dt)/(dlnA/dt). The E of the adsorbed PAA film was estimated during the PAA adsorption from the natural perturbation caused by minute variations in ambient temperature. 

### 3.1. Dynamic ST

**Figure 2:**
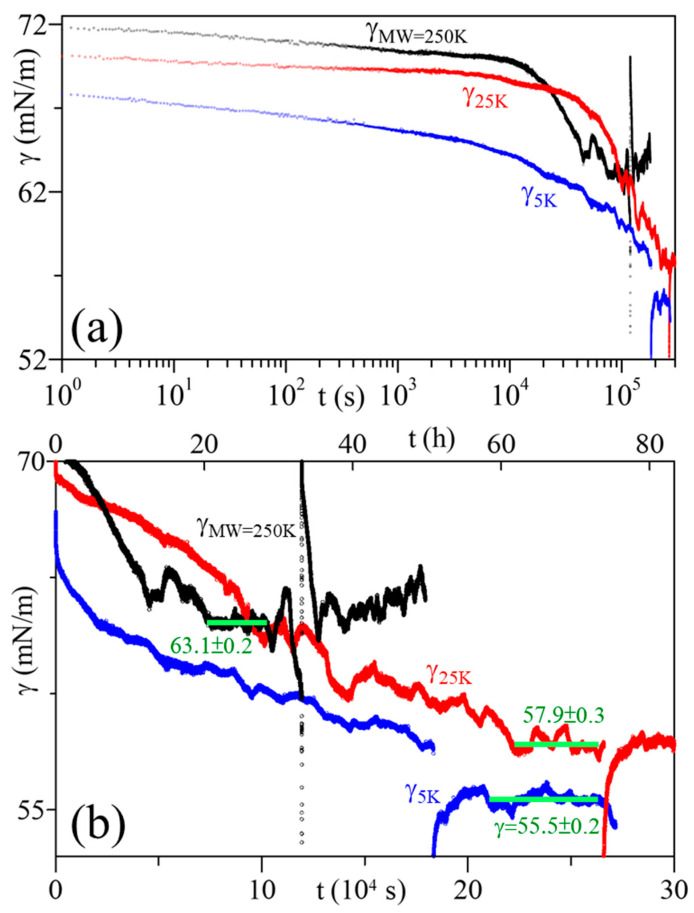
Surface tension (γ) relaxations of PAA solutions at C_PAA_ = 5 × 10^−4^ g/cm^3^; MW = 5, 25, 250 (kDa), time axis in: (**a**) log scale, and (**b**) linear scale. The horizontal lines denote the equilibrium-ST.

The ST relaxation profiles of PAA_(aq)_ solutions of three different MW are shown in Figure 2. For the solution of MW 250 kDa, the first ST data point was 71.6 mN/m, then ST decreased smoothly ~0.7 mN/m in the first ~100 s (Figure 2a). Later a smooth ST decrease was observed at a gradually slower rate, while the SA exhibited only small oscillations (amplitude ∆A/A~1%) at 1.5 h < t < 8 h (Figure S6b). As the adsorption progressed, both smaller oscillations (∆A/A~1%) and larger oscillations (∆A/A~4–6%) were detected (Figure S6c,d). However, the ST remained fairly constant for ~7 h in the later stage (t~21–28 h, Figure 2b), and it was thus set to be its equilibrium-ST. An illustration of these smaller and larger oscillations of the ST and SA relaxations in the earlier and later stages of the adsorption process is detailed in Figure S6. 

A similar relaxation and oscillation trend in ST and SA was also observed in the other two solutions with MW 5 and 25 (kDa). The ST decreased smoothly initially, then continued decreasing at a slower rate until eventually reaching a constant value, as the SA exhibited only small oscillations in the earlier stage, and both smaller and larger oscillations in the later stage (Figures S7–S9). However, the first ST data points were 67.8 and 70.1 (mN/m) for MW 5 and 25 (kDa), respectively (Figure 2a), compared to 71.6 mN/m for MW 250 kDa. Moreover, a significantly longer time (>55 h for both 5 and 25 kDa solutions) was needed to reach their constant ST (Figure 2b, Figures S10 and S11). 

### 3.2. Forced Perturbation

A forced perturbation of the pendant bubble was conducted at the later stage of the adsorption process to confirm whether the ST had indeed reached equilibrium or not. Figure 2b, Figures S12 and S13 illustrate a rapid compression at t = 26.576 (10^4^ s) for MW 25K and a rapid expansion at t = 11.930 for 250K. It was observed that the STs before and after perturbation are nearly the same in both cases. Note that the forced compression was performed too early (before it had reached its equilibrium-ST; Figure 2b and Figure S14) for the 5 kDa MW solution. Therefore, the forced compression accelerated the process of reaching its equilibrium-ST, resulting in a nearly constant ST after compression, ~3 mN/m lower than that before compression. Without this external compression, the solution would have needed a longer time to reach its equilibrium-ST. 

The data in Figure 2 also indicate that the PAA solution of lower MW takes a longer time to reach its equilibrium-ST. The data in Figure 2b, Figures S12 and S13 show that the forced perturbation can be used to confirm if the ST has reached equilibrium or not.

### 3.3. SA and ST Response to Temperature Variation

**Figure 3:**
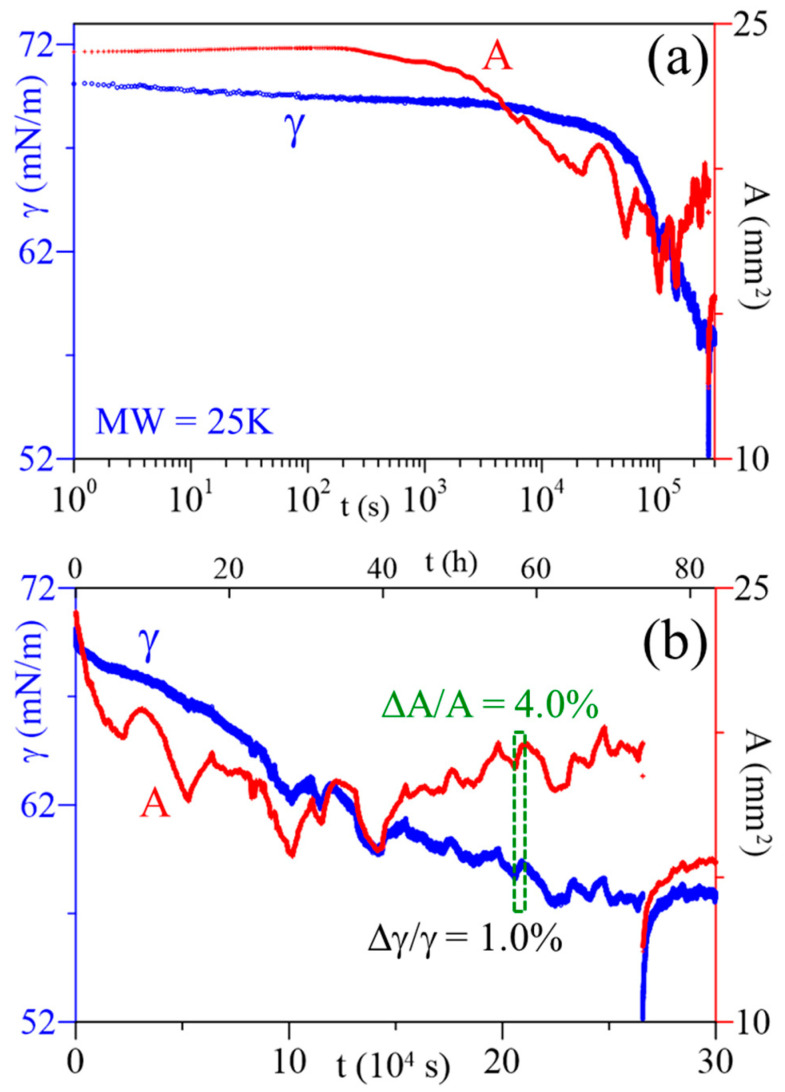
Relaxations of surface tension (γ) and surface area (A) of a PAA solution at C_PAA_ = 5 × 10^−4^ g/cm^3^, MW = 25 kDa, time axis in: (**a**) log scale, and (**b**) linear scale.

Figure 3 illustrates the relaxations of ST and bubble SA for a PAA solution of MW 25 kDa. The data indicate that the SA is more responsive to the temperature variation compared to the ST. In the earlier stage of the adsorption process (t < 6 h), the ST was decreasing smoothly and no significant perturbance (as a response to temperature variation) was observed, while the SA exhibited some oscillations with ∆A/A~1% (Figure S7b). As the adsorption progressed (t > 15 h, Figure S7c), some oscillations with ∆A/A~5% were detected, but the corresponding ST response was still smaller than that of SA (∆γ/γ < ∆A/A). Figure 3b illustrates an example where ∆A/A = 4%, and ∆γ/γ = 1% at t = 20.628–20.901 (10^4^ s). More examples of the solutions of MW 5 and 250 (kDa) are given in Figures S6, S8 and S9 and Table S3.

### 3.4. Equilibrium ST

**Figure 4:**
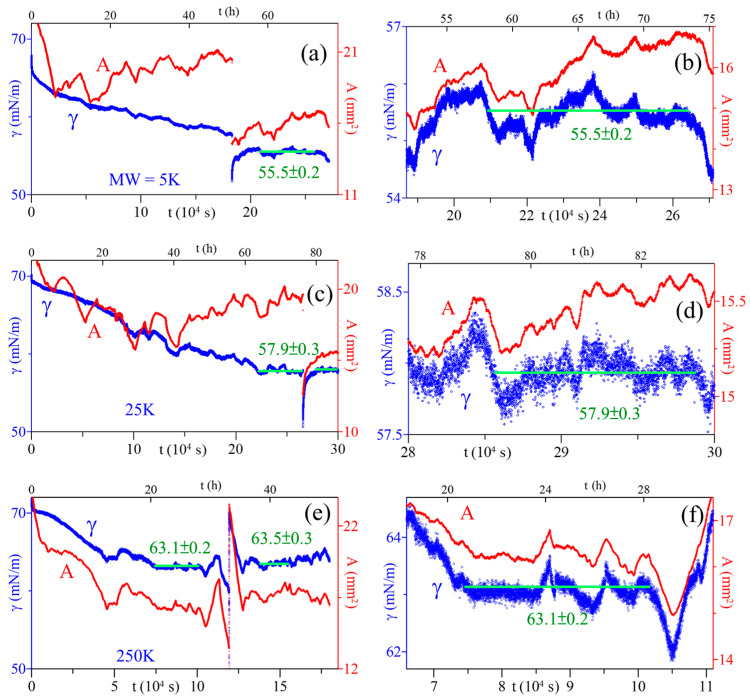
Relaxations of surface tension (γ) and surface area (A) of PAA solutions at C_PAA_ = 5 × 10^−4^ g/cm^3^, MW = 5 (**a**,**b**), 25 (**c**,**d**), 250 (**e**,**f**) (kDa). The horizontal lines denote the equilibrium-ST.

**Figure 5:**
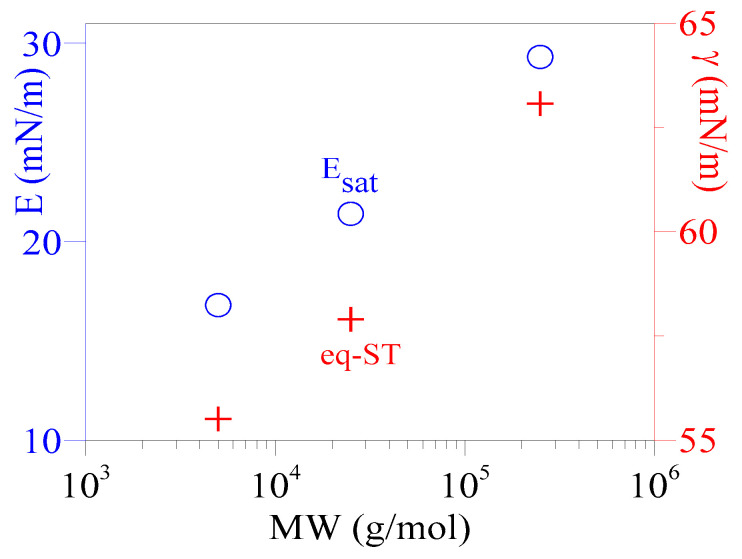
Variation of the equilibrium-ST of PAA solutions and the dilational modulus of saturated adsorbed film (E_sat_) as a function of PAA MW.

The ST relaxation data of PAA solutions in the later stage of the adsorption process were carefully examined. There exists a time interval, typically lasting several hours (8–15 h), where the ST for each PAA solution was essentially constant (with only slight fluctuations). The ST in this period, marked with horizontal green lines in Figure 2b, Figure 4, Figures S10 and S11, was set as the equilibrium-ST. For instance, the ST for the 5 kDa solution remained constant over 14 h (t~58–73 h) at 55.5 ± 0.2 mN/m. In comparison, the 25 and 250 kDa solutions have equilibrium-ST of 57.9 ± 0.3 and 63.1 ± 0.2 (mN/m), respectively. The equilibrium-ST data are plotted in Figure 5 as a function of PAA MW, showing that equilibrium-ST increases with polymer MW.

### 3.5. E_sat_ of Adsorbed PAA Films

**Figure 6:**
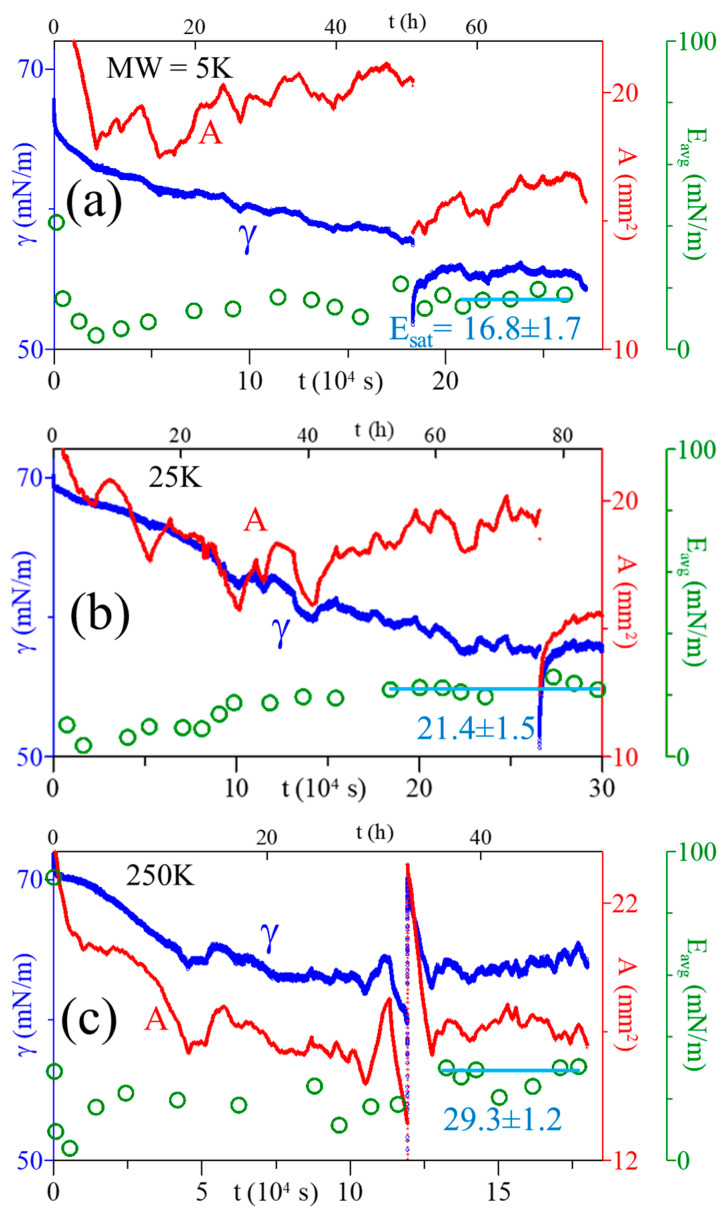
Variation of E_avg_ of purely aqueous PAA solutions alongside the corresponding relaxations of ST (γ) and SA (A) of the air bubbles at C_PAA_ = 5 × 10^−4^ g/cm^3^; MW = 5 (**a**), 25 (**b**), 250 (**c**) (kDa). The horizontal lines denote the E_sat_.

In this study, the relaxation data of ST and SA were used to evaluate the E_i_ (dilational modulus of one distinct perturbance) and E_avg_ (average dilational modulus of several consecutive perturbances over a much longer time) of the adsorbed PAA film throughout the adsorption process. Figure 6 shows the variations of E_avg_ (circles), along with the ST and SA, for three PAA solutions. In the early stage of the adsorption (t < 6 h), E_avg_ was observed to decrease with time until it reached a minimum of 4 mN/m for all three solutions. Subsequently, E_avg_ increased slowly as the ST relaxed (and the adsorbed film developed with time) and eventually leveled off, reaching a steady value (E_sat_, saturated dilational modulus of the adsorbed film). E_avg_ reached 29.3 ± 1.2 (mN/m) at t ≥ 36 h (the lifetime of the adsorbed film) for the PAA solution of 250 kDa; while it took >50 h and >58 h to reach 21.4 ± 1.5 and 16.8 ± 1.7 mN/m for solutions of 25 and 5 kDa, respectively (the horizontal lines in Figure 6 and Figure S15). The dependency of E_sat_ on PAA MW is shown in Figure 5, where E_sat_ increases with polymer MW. The data in Figure 6 also indicate that a significantly long duration is needed to reach the E_sat_ for PAA solutions. Moreover, a longer time is needed for a lower MW solution, similar to a longer time needed for a lower PAA MW solution to reach its equilibrium-ST. 

## 4. Discussion

**Figure 7:**
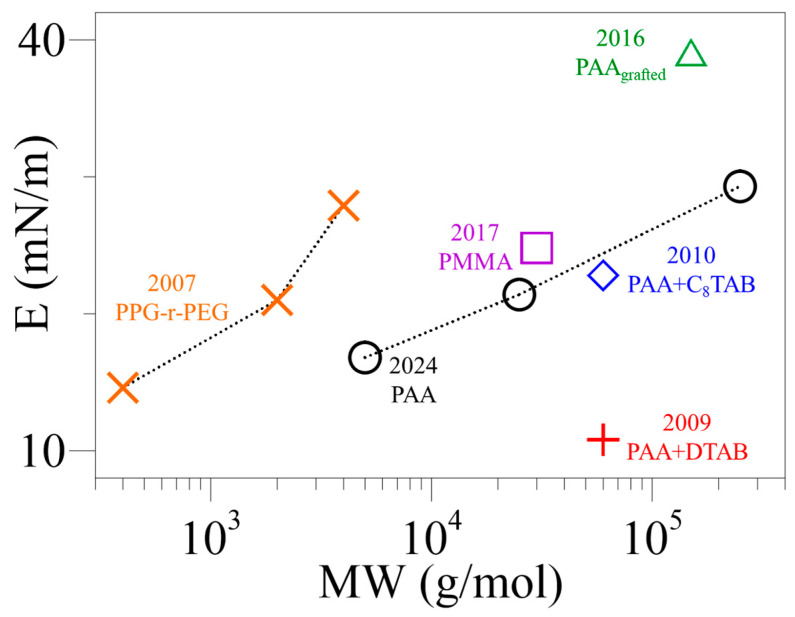
A comparison of the E/E_sat_ of polymer films reported in the literature and in this study.

The E_sat_ measured using this method was found to increase slightly with PAA MW. A comparison with the E of PAA + additive films reported in the literature was conducted and shown in Figure 7 and Figure S16, Table 3 and Table S4. Similar values, 10–40 mN/m, were obtained for the E or E_sat_ of PAA + additive films. However, E was found to increase at increasing salt or surfactant additive (concentration or chain length) [23,24,28], (Figure S16 and Table 2). The E of PPG-r-PEG films was also reported to increase with polymer MW; although at a lower polymer MW range [27]. In addition, the PMMA solution of MW 30 kDa shows a similar E, 25 mN/m [29].

In the early stage (t < 6 h) of the PAA adsorption, E_avg_ was detected to decrease with time and reached a minimum of ~4 mN/m. To understand this behavior, the variation of E_avg_ in globular protein solutions, BSA (bovine serum albumin) and HSA (human serum albumin), was examined [41,42,43]. All PAA, BSA and HSA solutions showed that E_avg_ decreased in the early stage, reached a minimum and then rose (Figure S17). We agree with what ref. [41] stated, that this “significantly higher E_avg_ was likely not real but rather due to the significant contribution of [PAA/protein] adsorption (which caused a significant decrease in ST)” at early adsorption.

The ST relaxation for PAA solutions was also examined and compared with globular protein (BSA and HSA) solutions of nearly the same wt% and MW. The data (Figures S18 and S19) show that the ST of a PAA solution relaxes slower than that of BSA and HSA solutions. 

Furthermore, a comparison of equilibrium-ST and E_sat_ of the adsorbed films of aqueous PAA, BSA and HSA solutions was conducted and shown in Figure S20: (i) the equilibrium-ST of PAA solutions is higher (55–63 mN/m) than that of BSA/HSA solutions (52–53 mN/m), and (ii) the adsorbed PAA film has a lower E_sat_ (17–30 mN/m) than that of globular protein films (~55 mN/m). Note that the E_sat_ for BSA and HSA solutions is a function of solution concentration [42,43]. The equilibrium-ST and E_sat_ data of other protein and PAA solutions at different wt% and MW are detailed in Table S5.

Further study is needed to improve the pendant bubble tensiometry method for evaluating the E of adsorbed polymer films. This could involve refining the experimental setup to reduce external factors impacting measurement accuracy, like temperature fluctuations. Exploring alternative analysis techniques, such as reflection IR spectrometry or X-ray and neutron reflectivity, might offer additional insights into PAA film molecular structure and behavior at the air–water interface. Investigating the effects of different environmental conditions, like varying pH levels or specific ions, on the E of PAA films could uncover further insights into the mechanisms governing their behavior. 

Additionally, understanding the influence of PAA’s polyelectrolytic behavior on its dilational rheology presents an intriguing avenue for future research. Investigating the extent of ionization of PAA and its consequent charge density at the air–water interface could shed light on the electrostatic interactions and intermolecular associations influencing the dilational modulus of adsorbed PAA films. Moreover, exploring the complex interplay between polymer structure, molecular weight, surface activity, and polyelectrolyte behavior would contribute to a more comprehensive understanding of PAA’s interfacial properties and behavior. These proposed research directions aim to expand on our study’s findings and clarify the complex interplay between polymer structure, molecular weight, surface activity, and interfacial behavior.

## 5. Conclusions

This study investigated the feasibility of evaluating the dilational modulus (E) of an adsorbed polymer film without subjecting a forced perturbation on the air–water interface. The relaxations of ST and bubble SA of PAA_(aq)_ solutions [MW = 5, 25, and 250 (kDa) at C = 5 × 10^−4^ g/cm^3^] were measured using a pendant bubble tensiometer throughout the PAA adsorption process. The relaxation data of ST and SA of distinct perturbances were used to evaluate the E_i_/E_avg_ of the adsorbed PAA film. Moreover, the variation of E_avg_ with time was also examined.

Slow adsorption of PAA film was observed, indicating a considerable time needed for reaching the equilibrium-ST and obtaining the E_sat_. The effect of MW on the ST relaxation, equilibrium-ST and E_sat_ of PAA solutions was examined: both the equilibrium-ST and E_sat_ were observed to increase with MW. This method, previously applied successfully to evaluate the E of adsorbed protein films, is shown to be workable for adsorbed PAA films.

## Figures and Tables

**Table 1:** Experimental conditions and key results in studies investigating the rheological properties of polymer films at an air–water interface.

Year [Ref.]	Compound			Instrument	ST	E
	Polymer ^1,2^	MW (kDa)	Conc.		t_life_ (h)	Result (mN/m)
			(10^−4^ g/cm^3^)			
2001 [35]	poly(DMAEMA-b-MMA)	42	-	SQELS, osc. ring	≤15	pH ↑→ E ↑ (10–25)
2004 [30]	PSS + DTAB	70	0.01, 0.05	osc. barrier + drop + Wilhelmy plate	≤8	C_surf_ ↑→ E ↓ then E ↑ (0–100)
2004 [31]	PSS + NaCl	70	500–1100	osc. barrier and Wilhelmy plate	≤5	C_NaCl_ ↑, C_pol_ ↑ → E ↑ (20–100)
2004 [33]	PNIPAM	300	0.09–1	osc. barrier	≤8	C_pol_ ↑→ E ↓ then E ↑ (50–60)
2007 [27]	PPG, copolymer (PPG + PEG)	PPG = 0.4, 2, 4, COP = 12	-	osc. barrier and Wilhelmy plate	-	MW ↑ → E ↑ (15–26)
2009 [28]	PAA, PMA + DTAB	60_(PAA)_, 100_(PMA)_	0.5	osc. ring	5	C_DTAB_ ↑ → E ↑ (5–50)
2010 [23]	PAA + C_n_TAB	60	0.5	osc. ring	5	C_CTAB_ ↑ → E ↑ (20–75)
2014 [25]	PNIPAM	46.7	-	osc. barrier and Wilhelmy plate	-	Π ↑→ E_max_ ↑, plateaus, then ↓ (40–60)
2014 [32]	PDADMAC/SDS/NaCl	100–200	0.1, 1.0	osc. barrier	≤5	C_surf_ ↑→ E ↓, then E ↑ (0–70)
2015 [26]	PEO_76_-PPO_29_-PEO_76_	8.35	0.19–0.48	osc. barrier and Wilhelmy plate	-	E_max_ (MW) (10–20)
2016 [24]	PAA (grafted)+ NaCl, HC1/C_10/12_-NH_2_	~150	7.5, 0.75	osc. bubble	~21	E (pH, additive), at γ_eq_ (35–60)
2017 [29]	modified PAA + PMMA	~30 (PMMA-AA)	0.03–300	osc. drop + bubble	~22	*f* ↑→ E ↑, at γ_eq_ (10–25)
2024	PAA	5, 25, 250	5	pendant bubble tensiometer	30+	MW ↑ → E_sat_ ↑, beyond γ_eq_ (16–30)

^1^ Poly(DMAEMA-b-MMA-poly((dimethylamino)ethyl methacracrylate-b-methyl methacrylate), PSS—poly(styrenesulfonate), PNIPAM—poly(N-isopropylacrylamide), PPG—poly(propylene glycol), PEG—poly(ethylene glycol), PMA—poly(methacrylic) acid, PDADMAC—poly(diallyldimethylammonium chloride), SDS—sodium dodecyl sulfate, PEO—poly(ethylene oxide), PPO—poly(propylene oxide), PAA (grafted)—PAA with decylamine (C_10_-NH_2_) or dodecylamine (C_12_-NH_2_), modified PAA—block polymer with poly(methylmethacrylate) PMMA; ^2^ DTAB—dodecyltrimethyl ammonium bromide; CnTAB—alkyltrimethylammonium bromide.

**Table 2:** Data summary of seven perturbances at t~57–59 h for a PAA solution of MW 5 kDa (Figure S2).

	t_0_ (10^4^ s)	t_1_ (10^4^ s)	∆A/A_0_ (%)	∆γ (mN/m)	dlnA/dt (10^−5^ s^−1^)	dγ/dt (10^−4^ mN/m·s)	E_i_ (mN/m)
1	20.47	20.61	9.09	0.16	0.63	1.07	17
2	20.68	20.74	7.89	0.11	1.10	1.46	13
3	20.75	20.78	−8.10	−0.11	−2.81	−4.55	16
4	20.88	20.93	−14.0	−0.11	−3.02	−3.46	11
5	21.00	21.05	−12.7	−0.18	−2.21	−3.04	14
6	21.10	21.22	−16.1	−0.21	−1.10	−1.41	13
7	21.23	21.35	6.02	0.13	0.52	1.17	22

**Table 3:** A comparison of the reported E of polymer films (Figure 7).

[Ref.], Year	Compound/ MW (kDa)	Conc. (10^−4^ g/cm^3^)	Additive Conc. (mol/cm^3^)	E (mN/m)	Remarks
[27], 2007	PPG-r-PEG/ 0.4, 2, 4	-	-	15, 21, 28 at MW = 0.4, 2, 4 kDa	E_max_
[28], 2009	PAA + DTAB/60	0.5	1, 7, 8.6 (10^−5^)	11, 46, 51 at C_add._ = 1, 7, 8.6 10^−5^ mol/cm^3^	E at t = 5 h
[23], 2010	PAA + C*_n_*TAB/60 *n* = 8, 10, 12, 14, 16	0.5	4.0 × 10^−7^	23, 49, 69, 76, 70 at *n* = 8, 10, 12, 14, 16	E_max_
[24], 2016	Grafted PAA + NaCl/150	7.5	-	39	pH = 7.7
			5.0 × 10^−4^	62	pH = 5.9
			1.0 × 10^−4^	64	pH = 6.4
[29], 2017	Modified PAA (with PMMA)/30	0.15	-	25	-
2024	PAA/ 5, 25, 250	5	-	17, 21, 29 at MW = 5, 25, 250 kDa	E_sat_

## Data Availability

Data are contained within the article.

## Supplementary Materials

The following supporting information can be downloaded at: https://www.mdpi.com/article/10.3390/polym16101359/s1, Table S1: Experimental conditions and key results in studies investigating the rheological properties of polymer films at an air–water interface; Figure S1: A schematic illustration of the pendant bubble tensiometer (a) and the thermostatic air chamber (b), along with a photo depicting the three temperature probes inside the chamber (c); Figure S2: (a) Relaxations of ST (γ) and SA of a pendant bubble for a purely aqueous PAA solution at C_PAA_ = 5 × 10^−4^ g/cm^3^ (MW 25 kDa). (b–d) Illustrations showing three additional perturbances with their respective linear fits for SA and ST; Figure S3: Relaxations of ST (γ) and SA of a purely aqueous PAA solution at C_PAA_ = 5 × 10^−4^ g/cm^3^ (MW = 5 kDa) at t = 0–28 (a,b), and 20.3–22.4 (c) (10^4^ s). Labels 1–7 denote the distinct perturbances identified at t = 20.47–21.35 (10^4^ s), and these seven data points were plotted (d), dγ/dt vs. dlnA/dt, to obtain the slope (E_avg_) of the best-fit; Table S2: Data summary of perturbances identified in Figure S3; Figure S4: Relaxations of ST (γ) and SA of a purely aqueous PAA solution at C_PAA_ = 5 × 10^−4^ g/cm^3^ (MW = 25 kDa) at t = 0–30 (a,b), and 17.0–19.5 (c) (10^4^ s). Labels 1–8 denote the distinct perturbances identified at t = 17.69–18.68 (10^4^ s), and these eight data points were plotted (d), dγ/dt vs. dlnA/dt, to obtain the slope (E_avg_) of the best-fit; Figure S5: Relaxations of ST (γ) and SA of a purely aqueous PAA solution at C_PAA_ = 5 × 10^−4^ g/cm^3^ (MW = 250 kDa) at t = 0–18 (a,b), and 17.2–17.96 (c) (10^4^ s). Labels 1–8 denote the distinct perturbances identified at t = 17.46–17.94 (10^4^ s), and these eight data points were plotted (d), dγ/dt vs. dlnA/dt, to obtain the slope (E_avg_) of the best-fit; Figure S6: (a) Relaxations of surface tension (γ) and bubble surface area of a PAA solution at C_PAA_ = 5 × 10^−4^ g/cm^3^, MW = 250 kDa. Regions show: (b) only smaller oscillations in SA (∆A/A~1%) during the earlier stage of adsorption; (c,d) both smaller and larger oscillations in SA (∆A/A > 4%) and ST in the later stage; Figure S7: (a) Relaxations of surface tension (γ) and bubble surface area of a PAA solution at C_PAA_ = 5 × 10^−4^ g/cm^3^, MW = 25 kDa. Regions show: (b) only smaller oscillations in SA (∆A/A~1%) during the earlier stage of the adsorption process; (c,d) both smaller and larger oscillations in SA (∆A/A > 2%) and ST in the later stage; Figure S8: (a) Relaxations of surface tension (γ) and bubble surface area of a PAA solution at C_PAA_ = 5 × 10^−4^ g/cm^3^, MW = 5 kDa. Regions show: (b) only smaller oscillations in SA (∆A/A~1%) during the earlier stage of the adsorption process; (c,d) both smaller and larger oscillations in SA (∆A/A > 2%) and ST in the later stage; Figure S9: Relaxations of surface tension (γ) and bubble surface area of PAA solutions at C_PAA_ = 5 × 10^−4^ g/cm^3^, MW = 5 (a,b), 25 (c,d), 250 (e,f) (kDa); Table S3: Summary of the responses in terms of percentage change in SA and ST at different stages of the adsorption for PAA solutions of MW = 5, 25, and 250 kDa (perturbances in Figures S6–S9); Figure S10: Relaxations of surface tension (γ) and surface area of PAA solutions at C_PAA_ = 5 × 10^−4^ g/cm^3^, MW = 5 (a), 25 (b), 250 (c) (kDa). The horizontal lines denote the equilibrium-ST; Figure S11. Relaxations of surface tension (γ) and bubble surface area of PAA solutions at C_PAA_ = 5 × 10^−4^ g/cm^3^, MW = 5, 25, 250 (kDa) at t = 6–28 (10^4^ s). The horizontal lines denote the equilibrium-ST; Figure S12: (a,b) Relaxations of ST (γ) and SA of a PAA solution at C_PAA_ = 5 × 10^−4^ g/cm^3^, MW = 25 kDa during a rapid perturbation (compression) of the pendant bubble; Figure S13: (a,b) Relaxations of ST (γ) and SA of a PAA solution at C_PAA_ = 5 × 10^−4^ g/cm^3^, MW = 250 kDa during a rapid perturbation (compression–expansion) of the pendant bubble; Figure S14: (a,b) Relaxations of ST (γ) and SA of a PAA solution at C_PAA_ = 5 × 10^−4^ g/cm^3^, MW = 5 kDa during the rapid perturbation (compression) of the pendant bubble; Figure S15: Variation of E_avg_ of PAA solutions alongside the corresponding relaxations of ST and bubble SA at C_PAA_ = 5 × 10^−4^ g/cm^3^, MW = 5 (a,b), 25 (c,d), 250 (e,f) (kDa). The horizontal lines denote the E_sat_; Figure S16: A comparison of the E/E_sat_ of polymer films reported in the literature and in this study; Table S4: Reported E of polymer films compared with the E_sat_ of PAA films in this study (Figure S16); Figure S17: Variation in E_avg_ (circles) of PAA and protein solutions alongside their corresponding relaxations of ST and bubble SA at: (a) C_BSA_ = 0.1 (mg/cm^3^); (b) C_HSA_ = 0.0027; (c) MW_PAA_ = 250 (kDa); and (d) MW_PAA_ = 5. The green boxes highlight the trend of E_avg_ observed in the early stage of adsorption; Table S5: Comparison of MW, concentration, eq-ST and E_sat_ for different protein/polymer solutions; Figure S18: Surface tension (γ) relaxations of PAA and protein solutions at: (a,b) C_BSA_ = 0.00035, 0.1, 0.4 (mg/cm^3^); (c,d) C_HSA_ = 0.00035, 0.1, 0.4; (e) MW_PAA_ = 5, 25, 250 (kDa); Figure S19: Surface tension (γ) relaxations of PAA and protein solutions of (a,b): MW_PAA_ = 5, 250 (kDa); MW_BSA_ = 66.4; and MW_HSA_ = 66.5; Figure S20: Variation of equilibrium-ST (a) of BSA/HSA/PAA solutions and the dilational modulus (E_sat_) of their saturated adsorbed films (b) as a function of MW.
