# Supplementary material for: A Study on the Dilational Modulus Measurement of Polyacrylic Acid Films at Air–Water Interface by Pendant Bubble Tensiometry
Source: Polymers (Basel). 2024 May 10;16(10):1359. doi: 10.3390/polym16101359 (PMC11125069; doi:10.3390/polym16101359)
Supplement: Supplementary file 1 [file polymers-16-01359-s001.zip › polymers-2886276-supplementary.pdf]

Supplementary Material

A Study on the Dilational Modulus Measurement of Polyacrylic Acid Films at Air–Water Interface by Pendant Bubble Tensiometry

Johann Eduardo Maradiaga Rivas [1], Li-Jen Chen [2], Shi-Yow Lin [1,*] and Siam Hussain [1,*]

[1] Department of Chemical Engineering, National Taiwan University of Science and Technology, 43, Sec. 4, Keelung Road, Taipei 106, Taiwan; m11006829@mail.ntust.edu.tw
[2] Department of Chemical Engineering, National Taiwan University, 1, Sec. 4, Roosevelt Road, Taipei City 106, Taiwan; ljchen@ntu.edu.tw
* Correspondence: sylin@mail.ntust.edu.tw (S.-Y.L.); d10806825@mail.ntust.edu.tw (S.H.); Tel.: +886-2-2737-6648 (S.-Y.L.)

S1. Literature Review—rheological properties of polymer films

A literature review, comparing the experimental conditions, solution composition and results of studies on the dilational rheology of adsorbed polymer films (listed in Table S1), was conducted, trying to find how the dilational modulus (E) is affected by different parameters. The E of polymer films was reported to be influenced by the polymer structure and solution composition (e.g. concentration, surface pressure, MW, additive, and pH). E was found to increase with increasing (i) surface pressure [1-3], (ii) polymer MW [1,3], (iii) salt concentration [4], and (iv) solution pH [5]. In addition, it was reported [3,4,6-9] that with an increasing concentration of polymer or surfactant, E initially decreases at low concentrations (10^{-5}–10^{-2} wt%), and then increases at higher concentrations (10^{-2}–10^{-1} wt%).

Most studies evaluate E from interfacial perturbations induced by an external force. Bykov et al. [8,10] utilized the oscillating ring method for examining the ST and E of solutions containing PMA/PAA+DTAB/C_nTAB. Aricov et al. [11] employed the oscillating bubble method to monitor the ST and E of PAA (grafted with decyl and dodecyl chains) solutions. Okumura and Kawaguchi [2] applied the oscillating barrier and Wilhelmy plate to evaluate the E of PNIPAM solutions. However, no study considered the potential effects of such external force-induced perturbations on the E measurement.

Moreover, the above reports generally did not specify the state of the adsorbed polymer films when E was evaluated, i.e., whether the solution had reached its equilibrium state, or whether the ST had reached its equilibrium value (γ_{eq}), even if some of the lifetimes of polymer films (t_{life}) was specified. The t_{life} at which E was measured was either 5–8 hours [4-6,8,10], 15–20 hours [5,11,12], or remained undisclosed [1-3]; but specifics on the state of the film are unclear. Diez-Pascual et al. [1] evaluated the E of PPG-r-

PEG films as a function of frequency and surface pressure, though the state of the adsorbed film was not stated at all.

Table S1. Experimental conditions and key results in studies investigating the rheological properties of polymer films at an air–water interface.

Ref	Year 1st author	Compound					Solvent	Instrument	ST	E		
		polymer[a]	MW (kDa)	conc. (10^{-4} g/cm^3)	additive/ modifier[b]	additive conc. mol/cm^3	pH/ buffer contents		t_{life} (h)	f (Hz)	ΔA/A (%)	Result (mN/m)
5	2001 Milling	poly(DMAEMA-b-MMA)	42	-	-	-	5-7.5	SQELS, osc. ring	≤ 15	-	-	**pH ↑ → E ↑** (10-25)
6	2004 Noskov	PSS	70	0.01, 0.05	DTAB	2×10^{-8}-10^{-4}	-	osc. barrier + drop + Wilhelmy plate	≤ 8	0.1-0.2	0.4-4	**C_{surf} ↑ → E↓ then E ↑** (0-100)
4	2004 Noskov	PSS	70	500-1100	NaCl	0-3×10^{-3}	-	osc. barrier and Wilhelmy plate	≤ 5	0.01-0.2	5	**C_{NaCl} ↑ , C_{pol} ↑ → E ↑** (20-100)
7	2004 Noskov	PNIPAM	300	0.09-1	-	-	-	osc. barrier	≤ 8	0.05-0.21	0.4-4	**C_{pol} ↑ → E↓ then E ↑** (50-60)
1	2007 Diez-Pascual	PPG, copolymer (PPG + PEG)	PPG = 0.4, 2, 4, COP = 12	-	-	-	-	osc. barrier and Wilhelmy plate	-	0.1	5	**MW ↑ → E ↑** (15-26)
10	2009 Bykov	PAA, PMA	60(PAA), 100(PMA)	0.5	DTAB	10^{-9}–10^{-5}	9.2/NaOH	osc. ring	5	0.1	7.4	**C_{DTAB} ↑ → E ↑** (5-50)
8	2010 Bykov	PAA	60	0.5	C_nTAB (n = 8 - 16)	1-4(10^{-7})	9.2/NaOH	osc. ring	5	0.1	7.4	**C_{CTAB} ↑ → E ↑** (20-75)
2	2014 Okumura	PNIPAM	46.7	-	-	-	/chloroform	osc. barrier and Wilhelmy plate	-	0.02	10	**Π ↑ → E_{max} ↑ , plateaus then ↓** (40-60)
9	2014 Lyadinskaya	PDADMAC /SDS/ NaCl	100-200	0.1, 1.0	SDS, NaCl	NaCl= 10^{-4} , SDS= 10^{-12}– 10^{-9}	-	osc. barrier	≤ 5	0.1	4	**C_{surf} ↑ → E↓, then E ↑** (0-70)
3	2015 Noskov	PEO_{76}-PPO_{29}-PEO_{76}	8.35	0.19-0.48	-	-	-	osc. barrier and Wilhelmy plate	-	0.14	5	**E_{max} (MW)** (10-20)
11	2016 Aricov	PAA (grafted)	~150	7.5, 0.75	NaCl, HC1/ $C_{10/12}$-NH_2	NaCl= 1-5(10^{-4})	3 - 9	osc. bubble	~21	0.01–0.2	-	**E (pH, additive), at γ_{eq}** (35-60)
12	2017 Gyurova	modified PAA	~30 (PMMA-AA)	0.03-300	PMMA	-	10	osc. drop + bubble	~22	0.005–0.2	-	**f ↑ → E ↑ , at γ_{eq}** (10-25)
	2024	PAA	5, 25, 250	5	-	-	-	pendant bubble tensiometer	30+	9×10^{-4} (avg)	0.25 (avg)	**MW ↑ → E_{sat} ↑ , beyond γ_{eq}** (16-30)

[a] **Poly(DMAEMA-b-MMA** - poly((dimethylamino)ethyl methacracrylate-b-methyl methacrylate), **PSS** - poly(styrenesulfonate), **PNIPAM**—poly(N-isopropylacrylamide), **PPG**—poly(propylene glycol), **PEG**—poly(ethylene glycol), **PMA**—poly(methacrylic) acid, **PDADMAC** - poly(diallyldimethylammonium chloride), **SDS** - sodium dodecyl sulfate, **PEO**—poly(ethylene oxide), **PPO**—poly(propylene oxide), **PAA (grafted)**—PAA with decylamine (C_{10}-NH_2) or dodecylamine (C_{12}-NH_2), **modified PAA—**block polymer with poly(methylmethacrylate) PMMA.

[b] **DTAB—**dodecyltrimethyl ammonium bromide; **C_nTAB**—alkyltrimethylammonium bromide.

S2. Apparatus

A pendant bubble tensiometer was employed to measure the ST relaxations of a globular protein solution; a schematic diagram and illustration are shown in Figure S1. This apparatus, utilized for generating a silhouette of the pendant bubble and its subsequent imaging and digitization, consists of the bubble-forming system, the imaging and recording system, and the video-image digitizer. The entire apparatus was contained in a fully enclosed room, maintained at 25±1°C, using a special air conditioner and heater.

The imaging and recording system is comprised of the following: (i) a halogen lamp (that serves as a light source, and emits light at a constant intensity) (ii) a planoconvex lens system (responsible for generating a collimated beam); (iii) an objective lens with an aperture of 7.1 (focal length = 60 mm); and (iv) a video camera. Note that the bubble's magnification on the camera's active area is approximately 1.5 times.

To create the bubble, a stainless steel needle (with an internal diameter = 0.41 mm) is utilized. This needle is connected to a miniature solenoid valve (manufactured by Lee Co.), which is linked to a Hamilton syringe in a Sage Instrument's syringe pump via Teflon tubing with an inner diameter of 1/16 inch (1.6 mm). The solenoid valve is controlled by a digital-to-analog (D/A) Data Translation card (model DT2801) installed on an IBM-PC AT computer. The video-image digitizer (DT 2861 Arithmetic Frame Grabber, Data Translation), also installed on the computer, digitizes the image into a resolution of 480 lines × 512 pixels and assigns each pixel an eight-bit grey level.

The environmental control chamber (made of acrylic material with 1 cm thickness) contained an adjustable stage inside. The adjustable stage was where the quartz cell with the aqueous surfactant/protein solution was placed. The environmental control chamber comprised a heater, a fan, and a cooling copper coil. The purpose of the cooling copper coil was to remove the heat by passing cooling water at a constant temperature. The heater was controlled by a computer with a PI control system in the following manner: the three thermistor probes sent temperature readings to a computer, and the computer, in turn, controlled the heat generator's power supply. During ST measurement, these three temperature probes (Figure S1c) monitored the ambient environmental temperature (air temperature T_{air} and solution temperature T_{sol}). The solution temperature probe was not immersed directly into the quartz cell but instead into a screw vial (near the quartz cell) containing DI water.

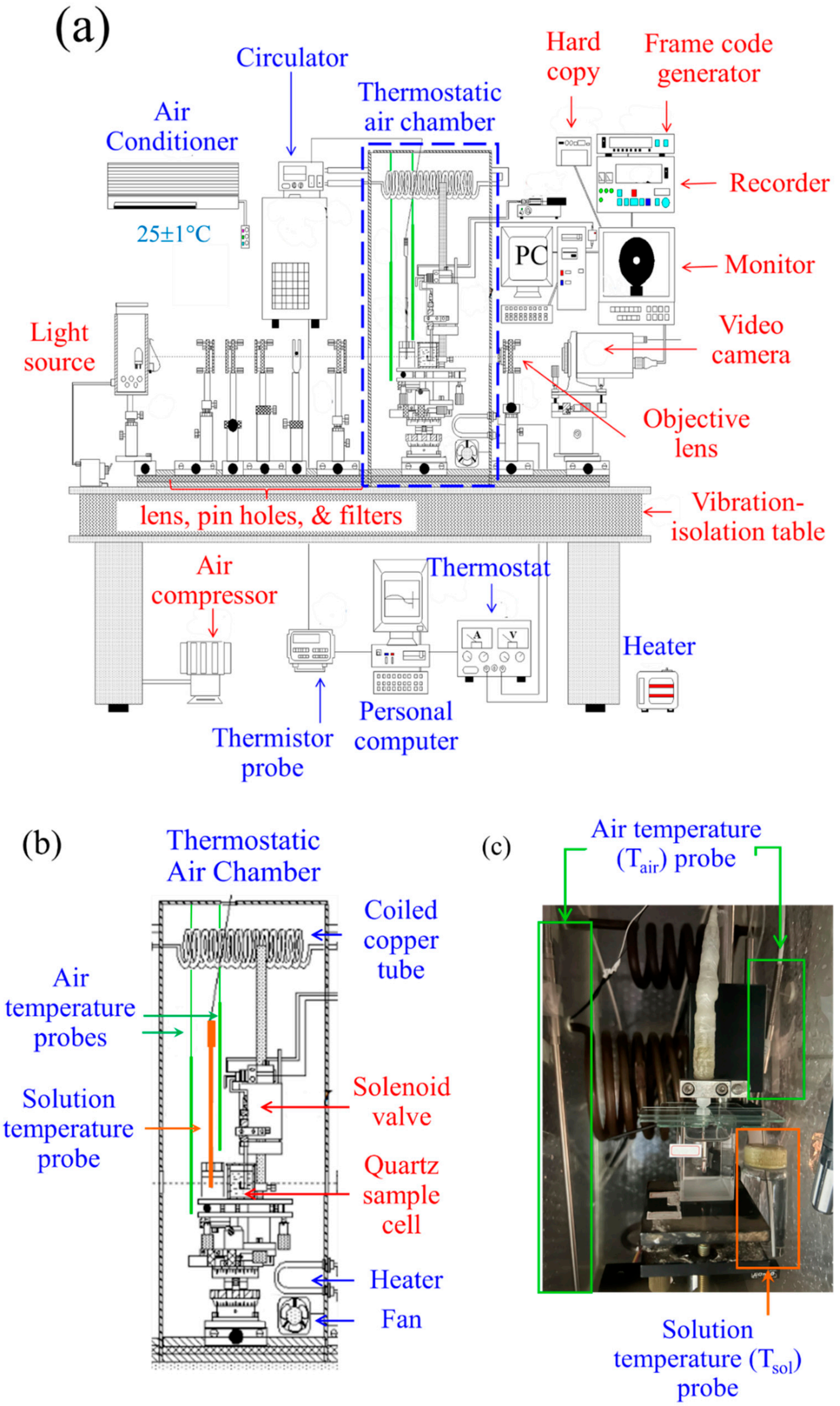

Figure S1. A schematic illustration of the pendant bubble tensiometer (a) and the thermostatic air chamber (b), along with a photo depicting the three temperature probes inside the chamber (c).

S3. Methodology—Evaluation of dilational modulus

The dilational modulus was evaluated following the manner in ref. [13]. This fitting process is applied throughout the entire SA and ST relaxation of each polymer solution studied and many E_i can be obtained for each ST perturbance. Three more examples are given in Figure S2 for C = 5×10^{-4} g/cm^3 (MW 25 kDa) at t = 8.197–8.247 (p1), 27.83–28.10 (p2), and 29.10–29.15 (p3) (10^4 s).

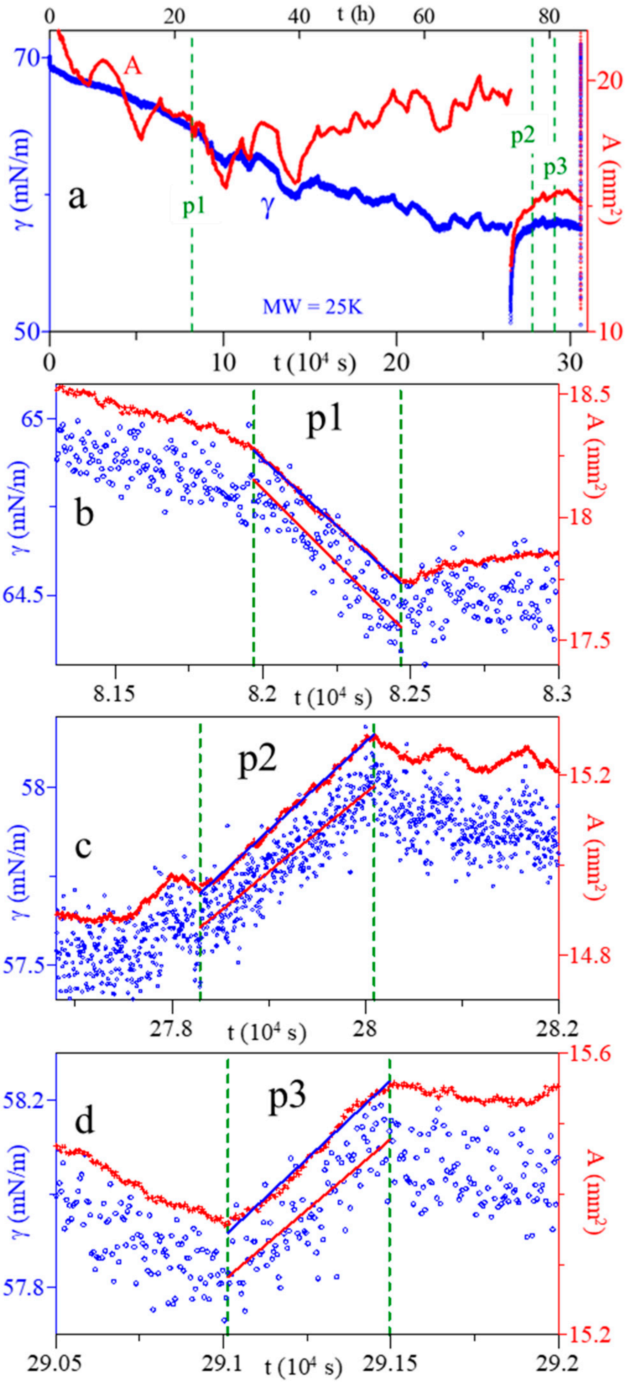

Figure S2. (a) Relaxations of ST (γ) and SA of a pendant bubble for a purely aqueous PAA solution at C_{PAA} = 5×10^{-4} g/cm^3 (MW 25 kDa). (b-d) Illustrations showing three additional perturbances with their respective linear fits for SA and ST.

The E_i (dilational modulus) was estimated from the surface dilational rate (dlnA/dt) and the rate of ST change (dγ/dt) of the bubble surface (E_i= dγ/dlnA) for every distinct perturbance. E_{avg} (average dilational modulus), was calculated from the slope of the best-fitting line in the plot between dγ/dt and dlnA/dt for several consecutive perturbances over a specific time range. Figure S3c illustrates seven individual perturbances at t = 20.47–21.35 (10^4 s) for a PAA solution of MW 5 kDa (E_i, i=1-7, green circles) and the raw data (t_0, t_1, A_0, A_1, $\Delta A/A_0$, γ_0, γ_1, $\Delta\gamma$, dlnA/dt, dγ/dt, and E_i) corresponding to perturbances 1–7 were tabulated in Table S2. Subsequently, the plot of dγ/dt vs. dlnA/dt was shown in Figure S3d), from which an E_{avg} (14 mN/m) at t = 20.47–21.35 (10^4 s) was obtained (shown as the horizontal line in Figure S3c). Two additional examples of the evaluation of E_{avg} are given in Figures S4-S5 for MW 25 and 250 kDa.

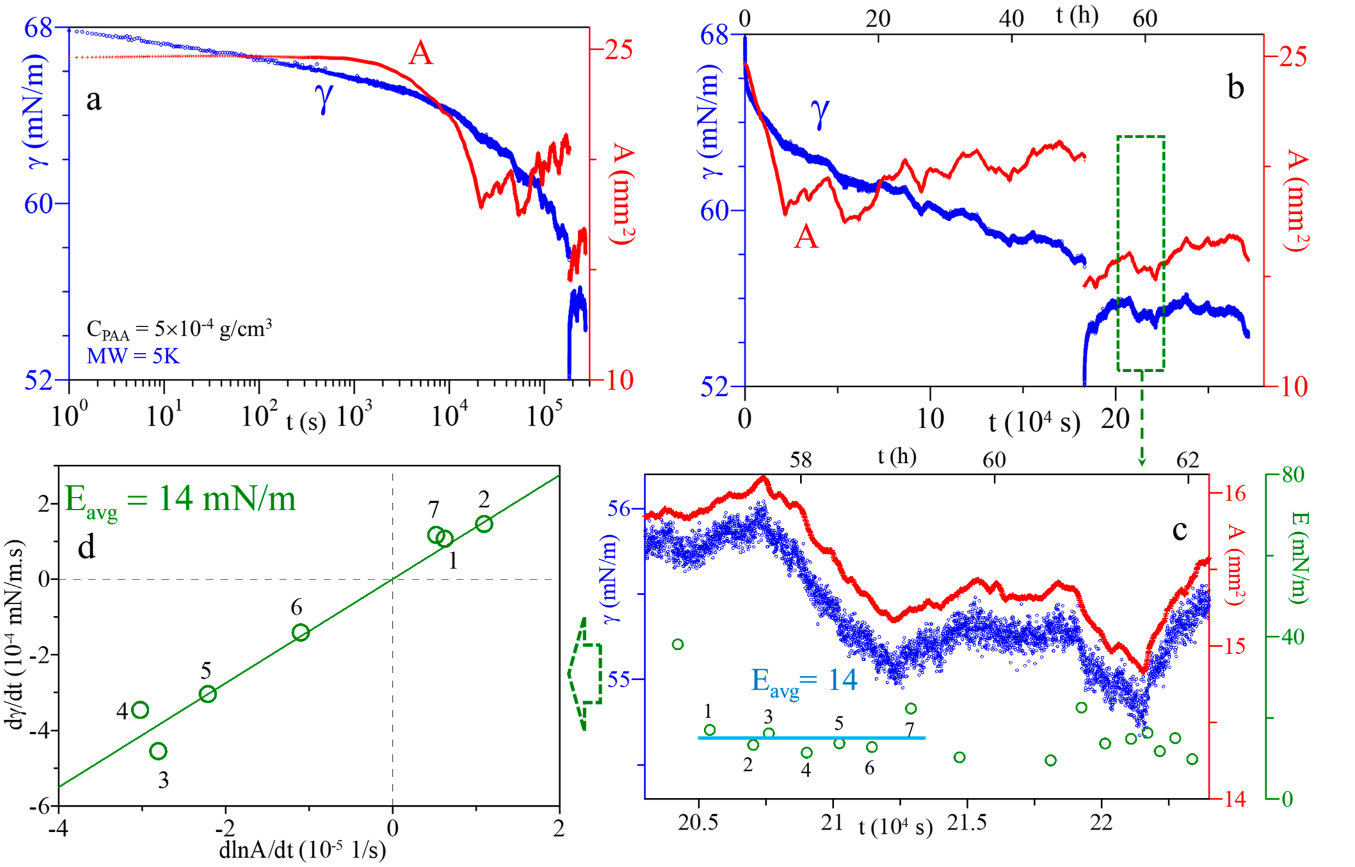

Figure S3. Relaxations of ST (γ) and SA of a purely aqueous PAA solution at C_{PAA} = 5×10^{-4} g/cm^3 (MW = 5 kDa) at t = 0–28 (a-b), and 20.3–22.4 (c) (10^4 s). Labels 1–7 denote the distinct perturbances identified at t = 20.47–21.35 (10^4 s), and these seven data points were plotted (d), dγ/dt vs. dlnA/dt, to obtain the slope (E_{avg}) of the best-fit.

Table S2. Data summary of perturbances identified in Figure S3.

	t_0 (10^4 s)	t_1 (10^4 s)	A_0 (mm^2)	A_1 (mm^2)	$\Delta A/A_0$ (%)	γ_0 (mN/m)	γ_1 (mN/m)	$\Delta\gamma$ (mN/m)	dlnA/dt (10^{-5} s^{-1})	dγ/dt (10^{-4} mN/m.s)	E_i (mN/m)
1	20.47	20.61	15.83	15.98	9.09	55.73	55.88	0.16	0.63	1.07	17
2	20.68	20.74	15.97	16.10	7.89	55.84	55.95	0.11	1.10	1.46	13
3	20.75	20.78	16.10	15.97	-8.10	55.94	55.81	-0.11	-2.81	-4.55	16
4	20.88	20.93	15.91	15.69	-14.0	55.69	55.53	-0.11	-3.02	-3.46	11
5	21.00	21.05	15.61	15.42	-12.7	55.44	55.26	-0.18	-2.21	-3.04	14
6	21.10	21.22	15.41	15.17	-16.1	55.29	55.08	-0.21	-1.10	-1.41	13
7	21.23	21.35	15.18	15.27	6.02	55.07	55.21	0.13	0.52	1.17	22

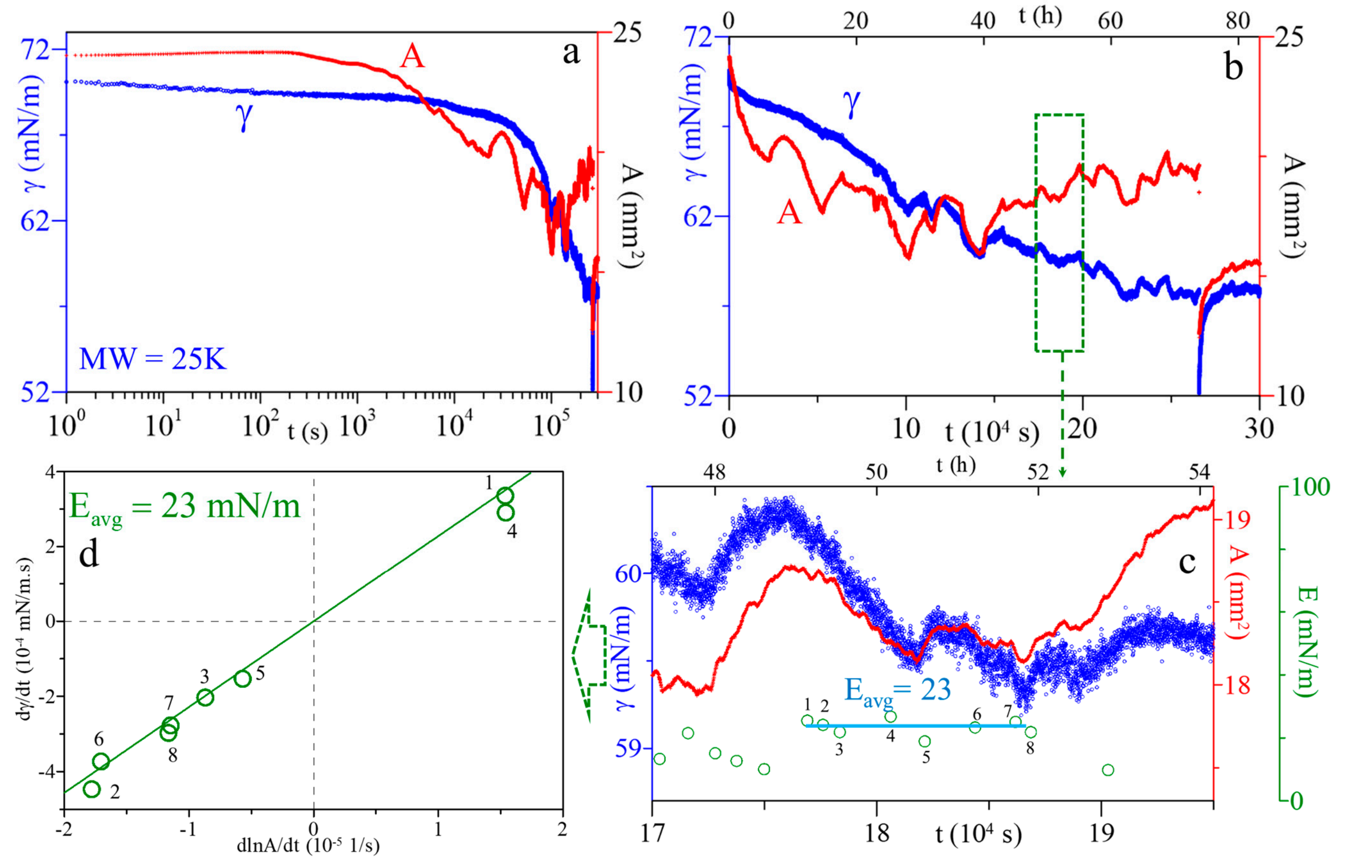

Figure S4. Relaxations of ST (γ) and SA of a purely aqueous PAA solution at $C_{PAA} = 5\times10^{-4}$ g/cm^3 (MW = 25 kDa) at t = 0–30 (a-b), and 17.0–19.5 (c) (10^4 s). Labels 1–8 denote the distinct perturbances identified at t = 17.69–18.68 (10^4 s), and these eight data points were plotted (d), dγ/dt vs. dlnA/dt, to obtain the slope (E_{avg}) of the best-fit.

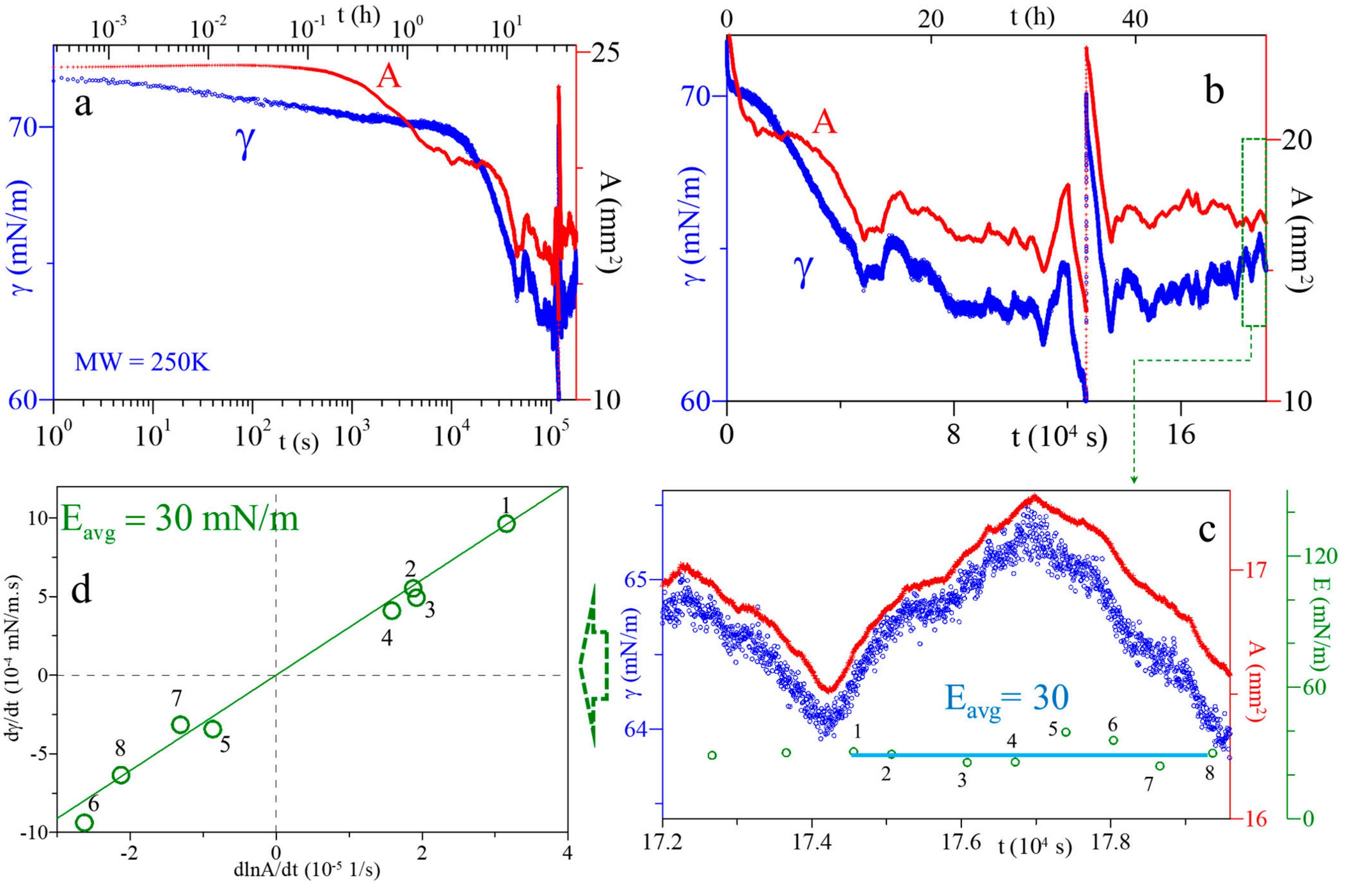

Figure S5. Relaxations of ST (γ) and SA of a purely aqueous PAA solution at C_{PAA} = 5×10^{-4} g/cm^3 (MW = 250 kDa) at t = 0–18 (a-b), and 17.2–17.96 (c) (10^4 s). Labels 1–8 denote the distinct perturbances identified at t = 17.46–17.94 (10^4 s), and these eight data points were plotted (d), dγ/dt vs. dlnA/dt, to obtain the slope (E_{avg}) of the best-fit.

S4. Dynamic ST of aqueous PAA solutions—oscillations in SA and ST

The ST along with the bubble SA relaxations for the PAA solution of MW 250 kDa are shown in Figure S6. The data indicate that the SA is more responsive to the temperature variation compared to the ST. Figure S6b shows the earlier stage of the relaxation at t = 0.5–3.0 (10^4 s), where the ST was decreasing smoothly and no significant perturbance (as a response to temperature variation) was observed, while the SA exhibited some oscillations with ΔA/A ~1%. As the adsorption progressed, at t = 4.5–7 (Figure S6c) and t = 14–18 (10^4 s) (Figure S6d), both smaller oscillations (ΔA/A ~1%) and larger oscillations (ΔA/A ~ 4–6%) were detected, but the corresponding ST response was still smaller than that of SA (Δγ/γ < ΔA/A). Recall that these oscillations are caused by minute variations in ambient temperature. A similar trend of relaxation and oscillation in ST and SA was also observed in the other solutions of MW 25 and 5 (kDa) (Figures S7-S8).

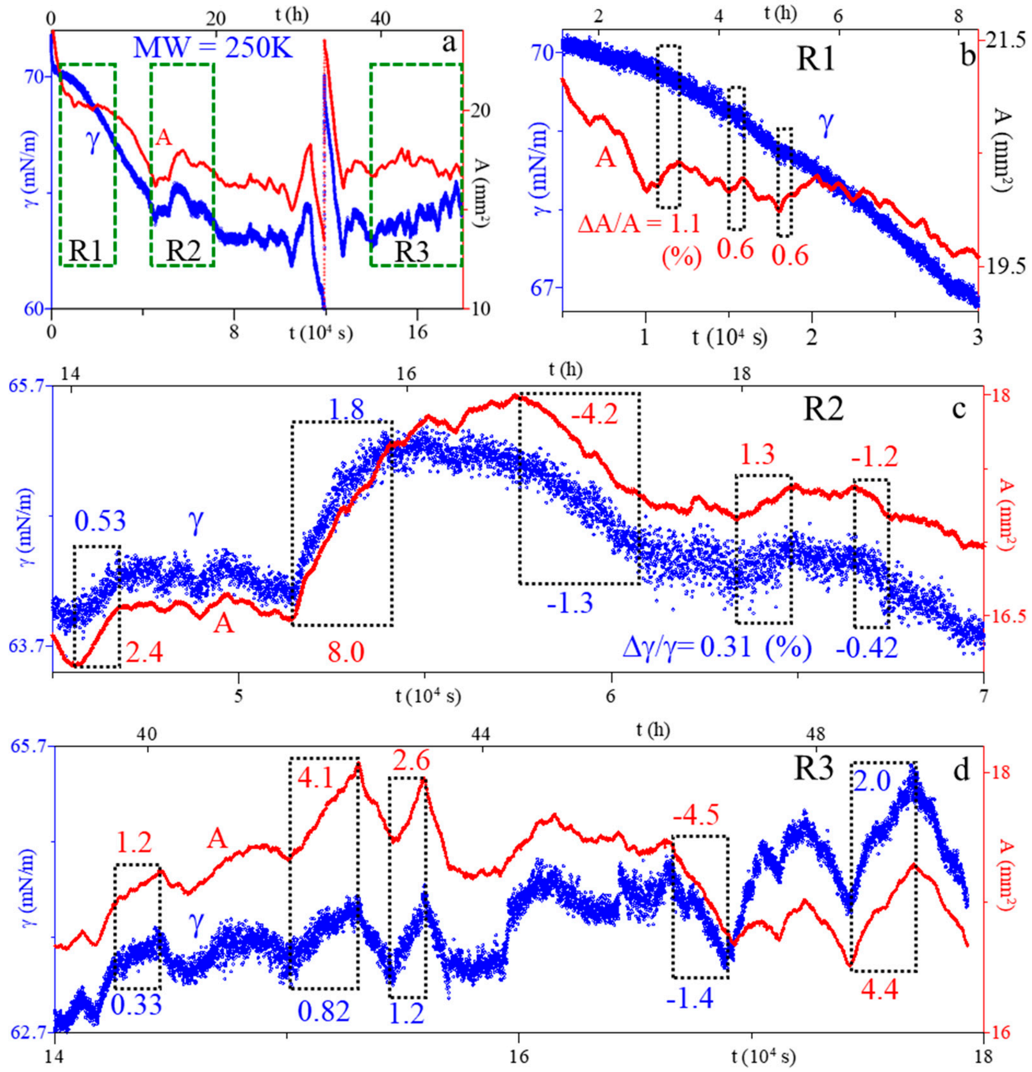

Figure S6. (a) Relaxations of surface tension (γ) and bubble surface area of a PAA solution at $C_{PAA} = 5\times10^{-4}$ g/cm^3, MW = 250 kDa. Regions show: (b) only smaller oscillations in SA (ΔA/A ~1%) during the earlier stage of adsorption; (c,d) both smaller and larger oscillations in SA (ΔA/A >4%) and ST in the later stage.

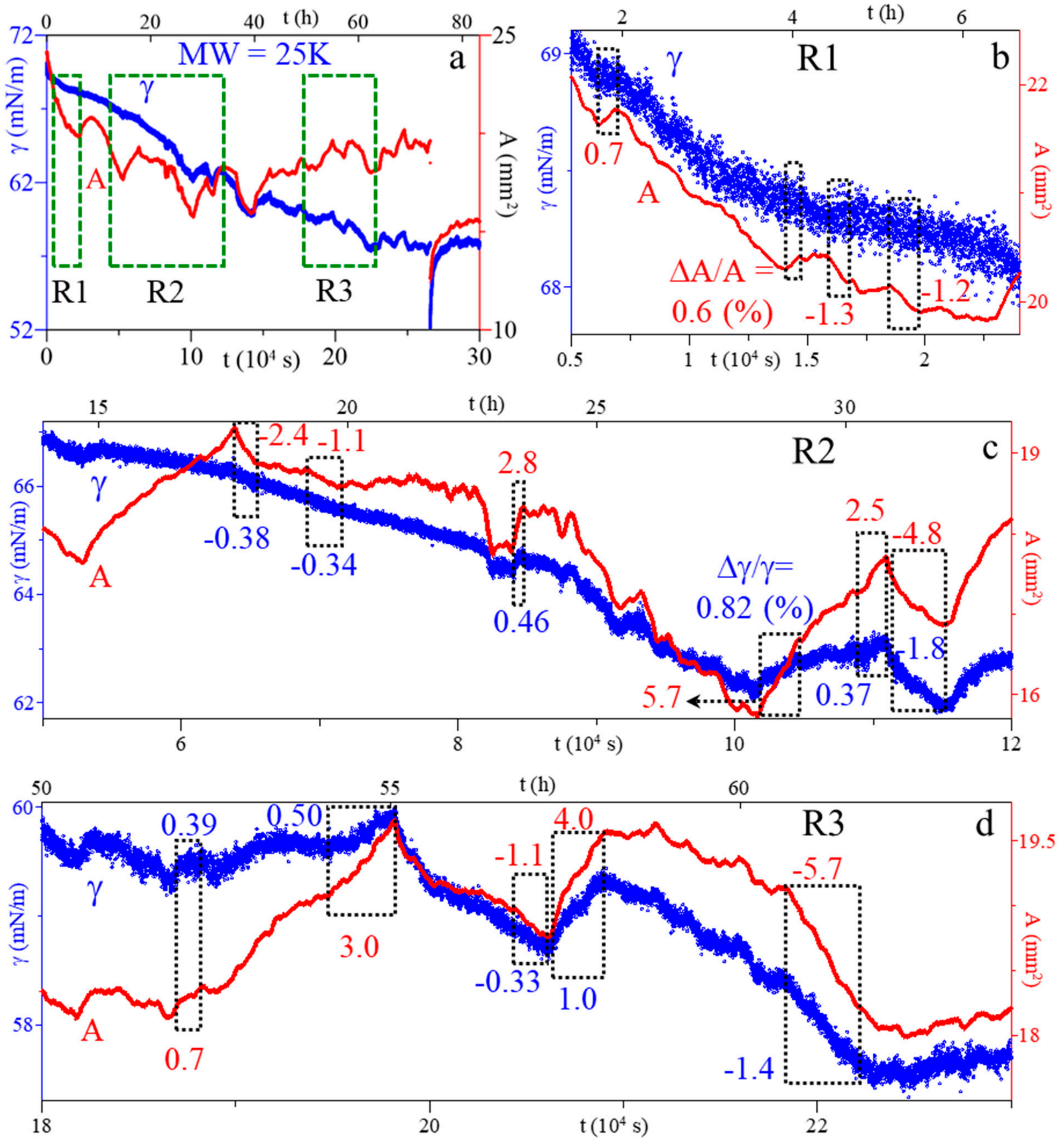

Figure S7. (a) Relaxations of surface tension (γ) and bubble surface area of a PAA solution at $C_{PAA} = 5\times10^{-4}$ g/cm^3, MW = 25 kDa. Regions show: (b) only smaller oscillations in SA (ΔA/A ~1%) during the earlier stage of the adsorption process; (c,d) both smaller and larger oscillations in SA (ΔA/A > 2%) and ST in the later stage.

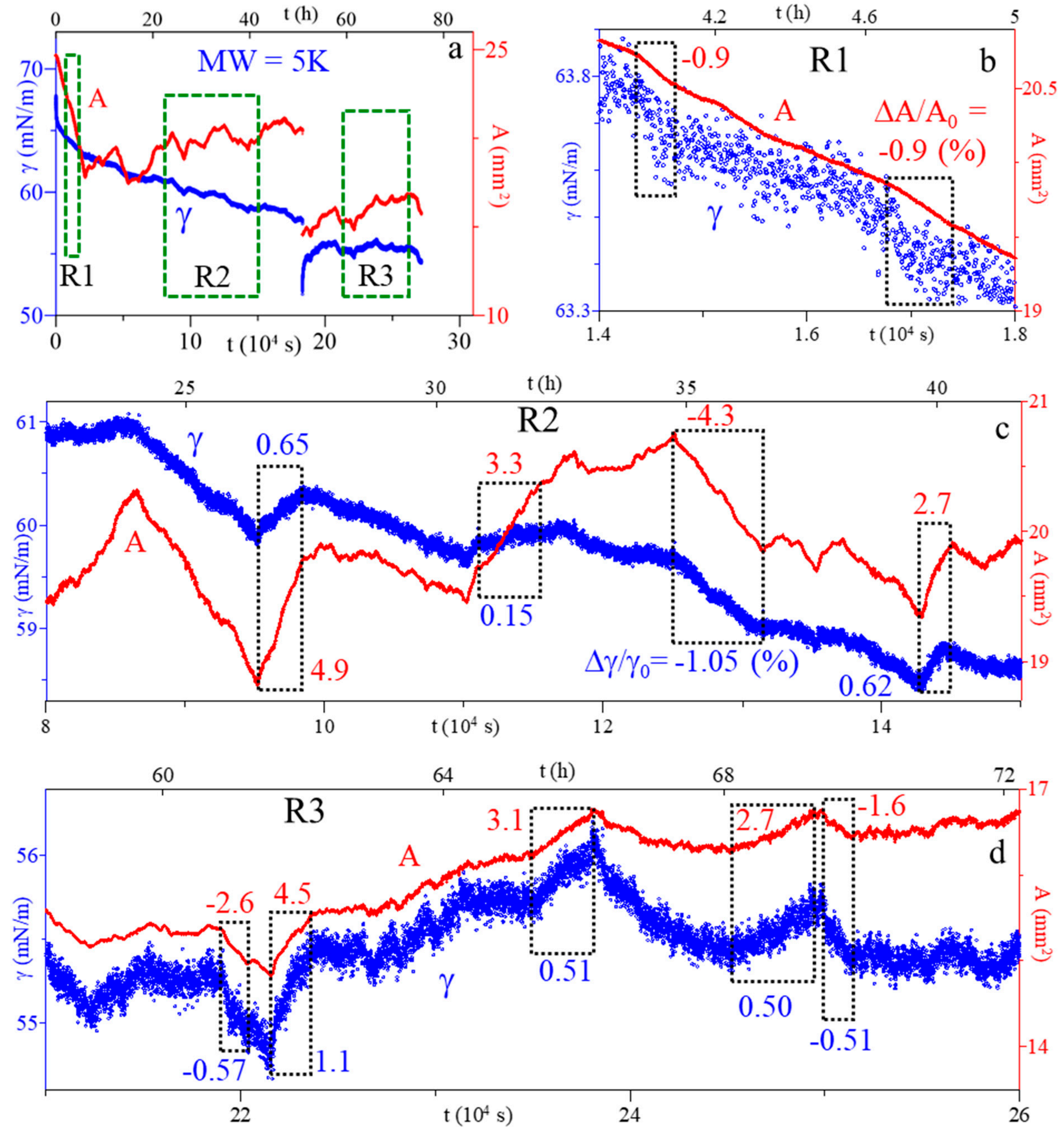

Figure S8. (a) Relaxations of surface tension (γ) and bubble surface area of a PAA solution at $C_{PAA} = 5\times10^{-4}$ g/cm^3, MW = 5 kDa. Regions show: (b) only smaller oscillations in SA (ΔA/A ~1%) during the earlier stage of the adsorption process; (c,d) both smaller and larger oscillations in SA (ΔA/A > 2%) and ST in the later stage.

The relaxations of ST and the bubble SA for PAA solutions of three different MW (5, 25 and 250 kDa) are plotted in Figure S9 for a comparison. For these three solutions, the SA always exhibits a larger response to the temperature variation compared to the ST during the adsorption. For example, at t = 9.54–10.01 (10^4 s) of the 5 kDa solution, the response of SA (ΔA/A) = 3.3% but the response of ST (Δγ/γ) = 0.15% only (Figure S9b). More examples of responses in ST and SA of these three PAA solutions are listed in Table S3.

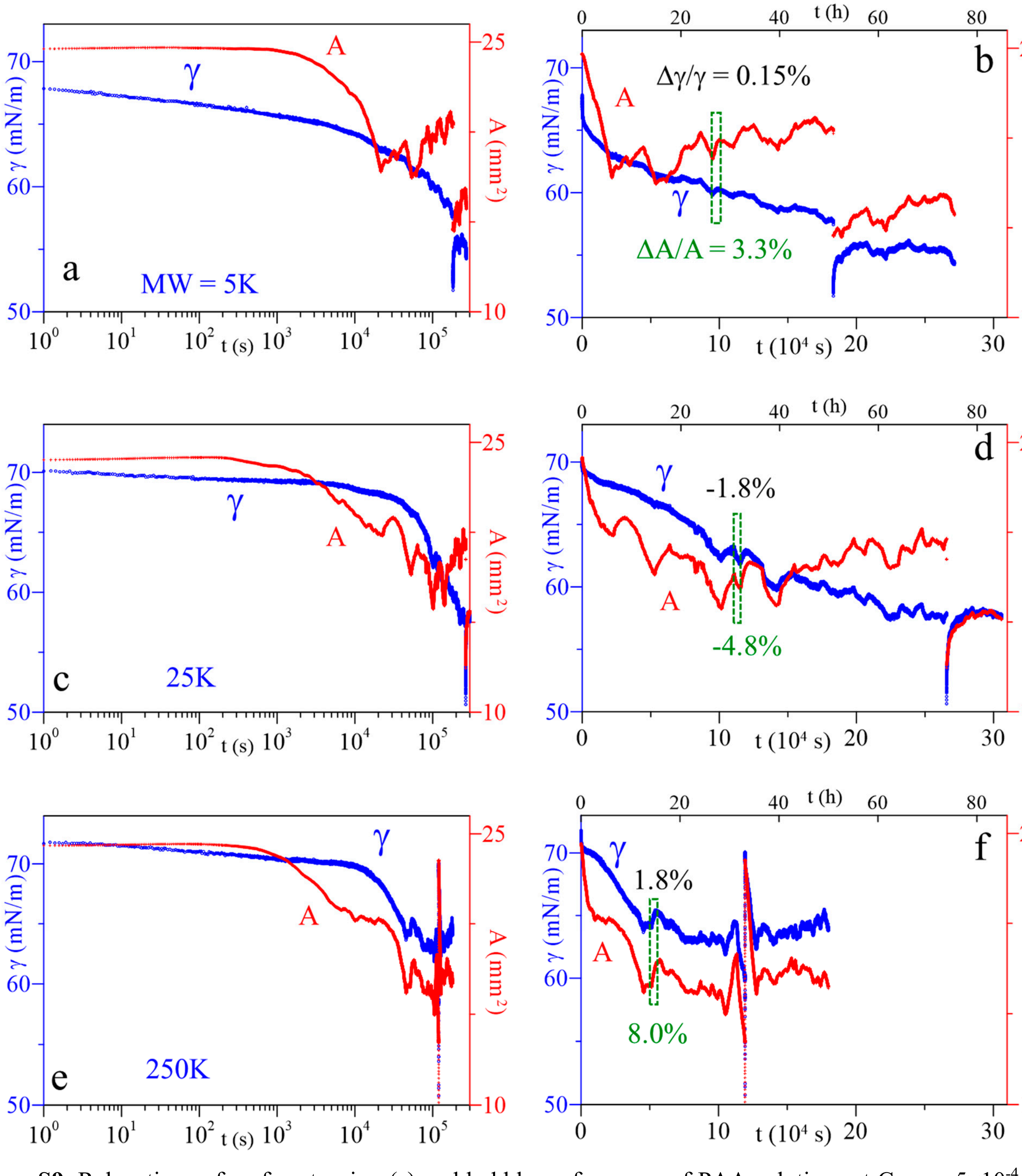

Figure S9. Relaxations of surface tension (γ) and bubble surface area of PAA solutions at C_{PAA} = 5×10^{-4} g/cm^3, MW = 5, (a-b), 25 (c-d), 250 (e-f) (kDa).

Table S3. Summary of the responses in terms of percentage change in SA and ST at different stages of the adsorption for PAA solutions of MW = 5, 25, and 250 kDa (perturbances in Figures S6-S9).

MW	Region	t_0 (10^4 s)	t_1 (10^4 s)	A_0 (mm^2)	A_1 (mm^2)	$\Delta A/A$ (%)	γ_0 (mN/m)	γ_1 (mN/m)	$\Delta\gamma/\gamma$ (%)	$\Delta A/A$ / $\Delta\gamma/\gamma$
5K	R1	1.438	1.469	20.72	20.54	-0.9	63.77	63.66	-	-
		1.679	1.721	19.85	19.67	-0.9	63.51	63.38	-	-
	R2	9.503	9.847	18.84	19.80	4.9	59.92	60.31	0.65	7.7
		11.092	11.548	19.72	20.38	3.3	59.82	59.9	0.15	22.2
		12.525	13.106	20.73	19.87	-4.3	59.63	59.00	-1.05	4.1
		14.267	14.479	19.35	19.89	2.7	58.44	58.8	0.62	4.4
	R3	21.906	22.029	15.37	14.98	-2.6	55.26	54.95	-0.57	4.5
		22.151	22.368	14.88	15.56	4.5	54.84	55.43	1.08	4.1
		23.518	23.810	16.24	16.76	3.1	55.74	56.03	0.51	6.1
		24.526	24.960	16.28	16.73	2.7	55.43	55.7	0.50	5.5
		24.988	25.131	16.73	16.46	-1.6	55.67	55.39	-0.51	3.2
25K	R1	0.617	0.684	21.63	21.77	0.7	68.91	68.88	-	-
		1.408	1.458	20.30	20.42	0.6	68.38	68.35	-	-
		1.584	1.662	20.43	20.17	-1.3	68.32	68.29	-	-
		1.852	1.985	20.14	19.89	-1.2	68.25	68.24	-	-
	R2	6.390	6.544	19.30	18.85	-2.4	66.19	65.94	-0.38	6.2
		6.919	7.129	18.79	18.59	-1.1	65.75	65.53	-0.34	3.2
		8.399	8.465	17.82	18.33	2.8	64.42	64.72	0.46	6.2
		10.161	10.459	15.75	16.68	5.7	62.22	62.73	0.82	7.0
		10.889	11.088	17.25	17.69	2.5	62.88	63.11	0.37	6.8
		11.098	11.518	17.71	16.88	-4.8	63.11	62	-1.77	2.7
	R3	18.666	18.715	18.14	18.27	0.7	59.32	59.55	0.39	1.8
		19.487	19.807	19.06	19.64	3.0	59.63	59.93	0.50	5.9
		20.429	20.590	18.98	18.78	-1.1	58.91	58.72	-0.33	3.2
		20.628	20.901	18.79	19.56	4.0	58.73	59.32	1.00	4.1
		21.853	22.315	19.13	18.07	-5.7	58.37	57.56	-1.40	4.1
250K	R1	1.082	1.200	20.21	20.43	1.1	69.77	69.63	-	-
		1.507	1.583	20.16	20.28	0.6	69.25	69.15	-	-
		1.805	1.863	20.01	20.13	0.6	67.99	68.20	-	-
		2.617	2.663	19.86	19.91	0.3	67.46	67.39	-0.10	2.9
		2.676	2.715	19.90	19.76	-0.7	67.38	67.25	-0.20	3.8
		2.847	2.931	19.59	19.65	0.3	67.00	66.97	-0.05	6.8
	R2	4.561	4.682	16.17	16.57	2.4	63.93	64.27	0.53	4.6
		5.142	5.396	16.48	17.85	8.0	64.13	65.26	1.80	4.5
		5.746	6.116	17.98	17.24	-4.2	65.13	64.32	-1.30	3.4
		6.340	6.484	17.16	17.38	1.3	64.25	64.45	0.31	4.1
		6.656	6.738	17.37	17.17	-1.2	64.40	64.14	-0.42	2.8
	R3	14.280	14.458	17.02	17.23	1.2	63.43	63.64	0.33	3.6
		15.021	15.279	17.33	18.06	4.1	63.44	63.97	0.82	5.0
		15.456	15.591	17.48	17.94	2.6	63.27	64.03	1.20	2.2
		16.658	16.891	17.47	16.70	-4.5	64.34	63.47	-1.37	3.3
		17.424	17.699	16.53	17.27	4.4	64.03	65.34	2.00	2.1

S5. Equilibrium ST of aqueous PAA solutions

The ST data for each PAA solution show essentially constant (with only slight fluctuations) for several hours (8–15 h). The ST in this period, marked with horizontal green lines in Figures S10 and S11, was set as the equilibrium-ST (γ_{eq}). The ST for the 5 kDa solution remained constant over 14 h (t ~ 58–73 h) at 55.5±0.2 mN/m (Figure S10a), while γ_{eq} for the solution of 25 kDa was found to be

57.9±0.3 for ~10 h (t ~ 62–72 h) before the bubble compression, and for ~4 h (t ~ 79–83 h) after the bubble compression (Figure S10b). For the 250 kDa solution, γ_{eq} = 63.1±0.2 mN/m for ~7 h (t ~ 21–28 h, Figure S10c) before the forced perturbation, and for ~5 h (t ~ 38–43 h) after the bubble expansion.

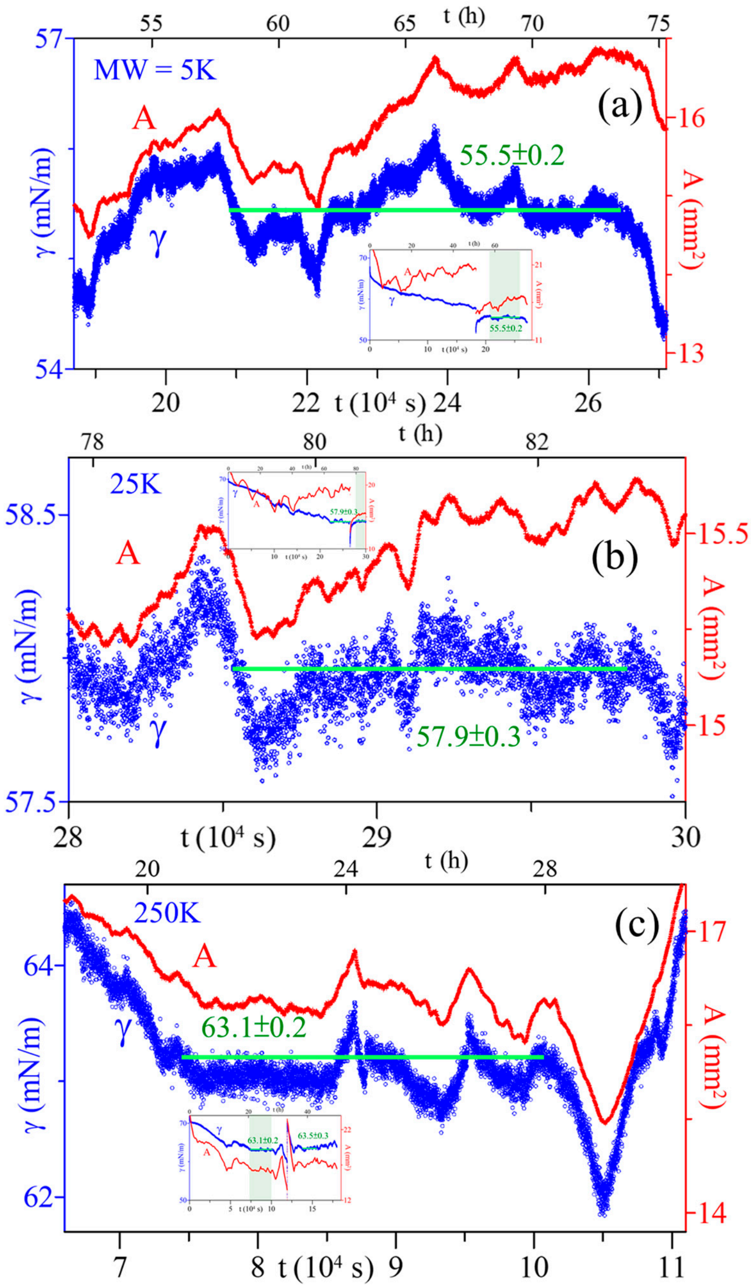

Figure S10. Relaxations of surface tension (γ) and surface area of PAA solutions at C_{PAA} = 5×10^{-4} g/cm^3, MW = 5 (a), 25 (b), 250 (c) (kDa). The horizontal lines denote the equilibrium-ST.

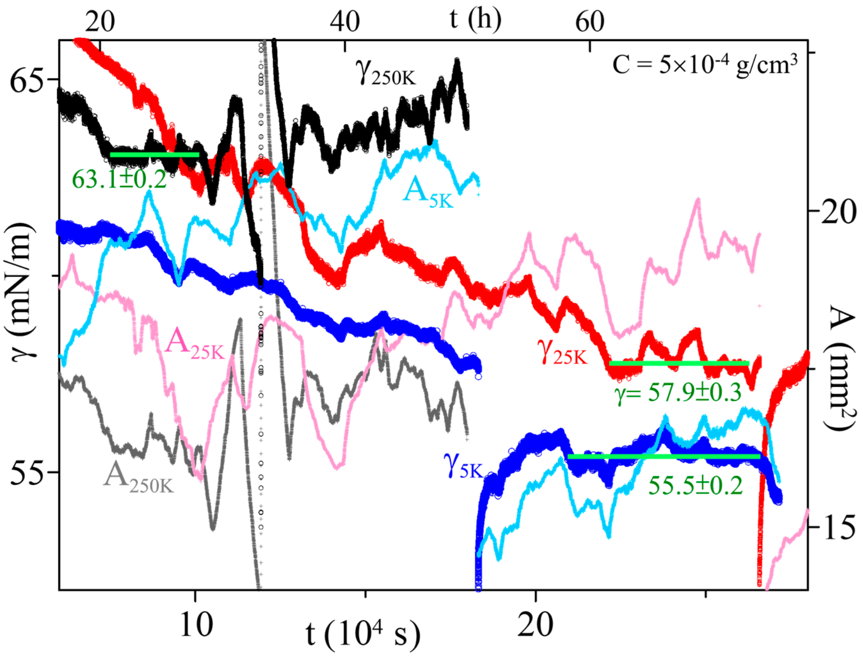

Figure S11. Relaxations of surface tension (γ) and bubble surface area of PAA solutions at C_{PAA} = 5×10^{-4} g/cm^3, MW = 5, 25, 250 (kDa) at t = 6–28 (10^4 s). The horizontal lines denote the equilibrium-ST.

A forced perturbation of the pendant bubble (either a rapid compression or expansion) was conducted at the later stage of the adsorption process to check whether the ST had indeed reached equilibrium or not. Figure S12 illustrates the forced compression, ~45% decrease in SA within ~0.7 s at t = 26.576 (10^4) s, for the PAA solution of MW 25 kDa. The ST dropped from 57.8 to 50.6 (mN/m), then gradually rose and eventually reached its previous γ_{eq} (~58 mN/m). Another forced perturbation (compression and then expansion) is shown in Figure S13 for the 250 kDa solution at t = 11.930 (10^4 s), with an initial ~23% SA decrease in ~0.1 s, then an abrupt ~150% increase in ~7 s (Figure S13b). During this perturbation, the ST first dropped from 58.7 to 46.3, then increased to 70.1 (mN/m), after a few hours the ST eventually relaxed back to its γ_{eq} (63.5 mN/m).

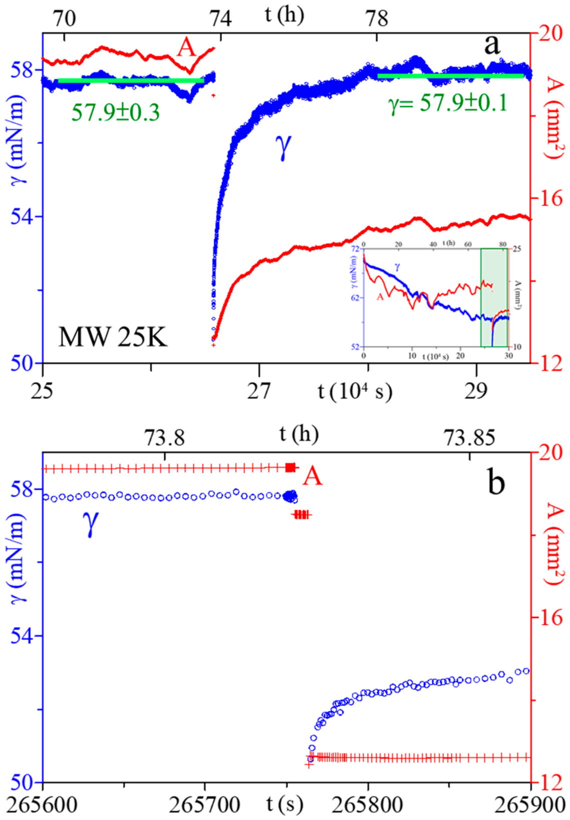

Figure S12. (a-b) Relaxations of ST (γ) and SA of a PAA solution at C_{PAA} = 5×10^{-4} g/cm^3, MW = 25 kDa during a rapid perturbation (compression) of the pendant bubble.

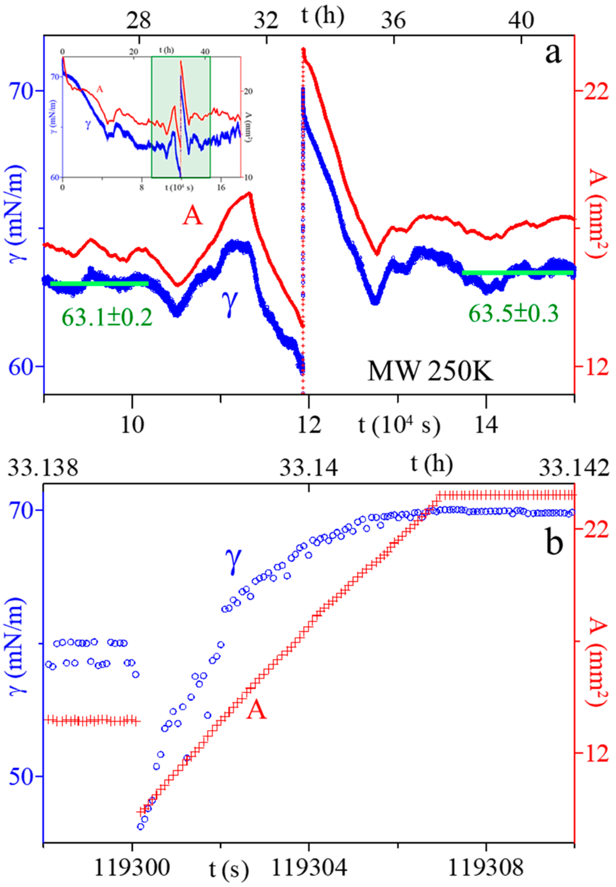

Figure S13. (a-b) Relaxations of ST (γ) and SA of a PAA solution at C_{PAA} = 5×10^{-4} g/cm^3, MW = 250 kDa during a rapid perturbation (compression–expansion) of the pendant bubble.

For the PAA solution of MW 5 kDa, the forced perturbation (compression) was performed before the ST reached its equilibrium-ST (Figure S14). This forced compression accelerated the adsorption, resulting in reaching a nearly constant ST faster, which was ~3 mN/m lower than that before compression. Without this external compression, the solution would have needed a longer time to reach its γ_{eq}.

The ST for the 250 kDa solution took ~21 h to reach its γ_{eq}, while a significantly longer time was observed for both 25 (> 62 h) and 5 kDa (>> 58 h) solutions to reach their constant ST (Figures S10-S11). Note that the solution of MW 5 kDa reached γ_{eq} earlier in Figure S14 because the adsorption was accelerated by the forced compression before the equilibrium state. It is believed that the time for the 5 kDa solution to reach its γ_{eq} should be >> 58 h. Therefore, the data (Figures S10-S14) imply that a PAA solution of lower MW takes a longer time to reach its equilibrium-ST.

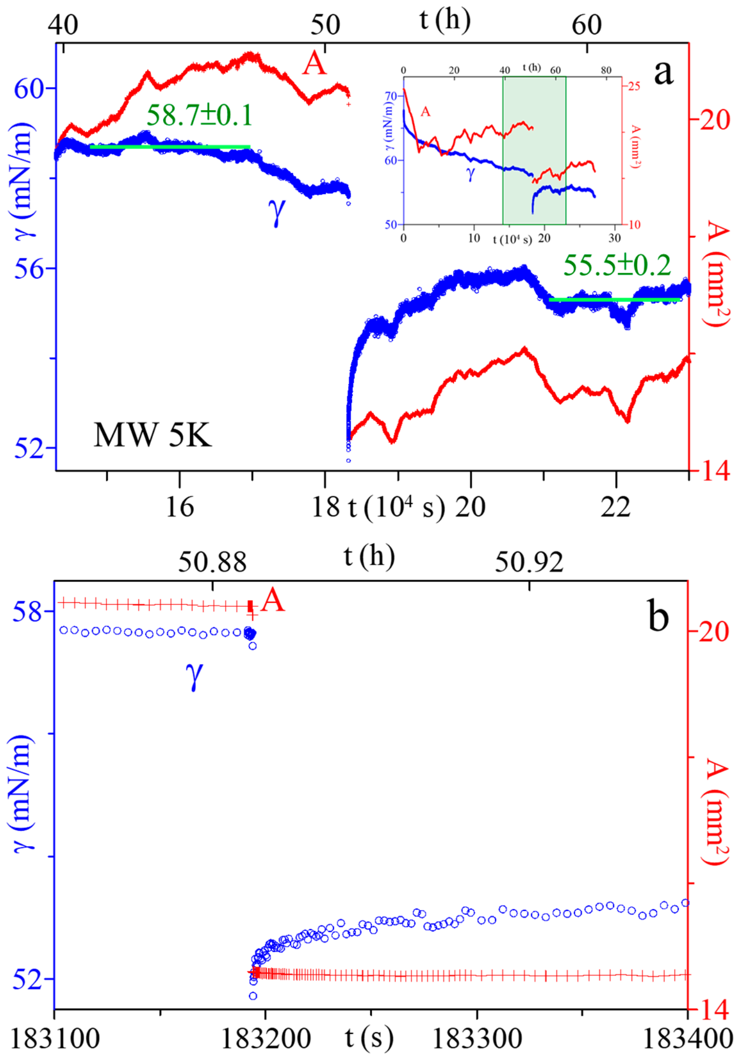

Figure S14. (a-b) Relaxations of ST (γ) and SA of a PAA solution at $C_{PAA} = 5\times10^{-4}$ g/cm^3, MW = 5 kDa during the rapid perturbation (compression) of the pendant bubble.

S6. E_{sat} of aqueous PAA solutions

The E_{avg} (average dilational modulus of several consecutive perturbances over a specific time range) increased slowly as the ST relaxed (during which the adsorbed film was developing with time) and eventually leveled off, reaching a steady value (E_{sat}, saturated dilational modulus of the adsorbed film). The variation of E_{avg} (green circles) along with the complete ST and SA relaxations for three PAA solutions at C = 5×10^{-4} g/cm^3 [MW = 5 (a), 25 (c) and 250 (e) (kDa)] are shown in Figure S15. E_{sat} can be obtained at the late stage of the adsorption for the above three PAA solutions: E_{avg} reached 16.8±1.7 (mN/m) at t ≥ 58 h for 5 kDa solution (Figure S15b); while it took > 50 h and > 36 h to reach 21.4±1.5 and 29.3±1.2 mN/m for solutions of 25 and 250 kDa (Figure S15d,f), respectively. The data indicate that a significantly long duration is needed for PAA solutions to reach their E_{sat}.

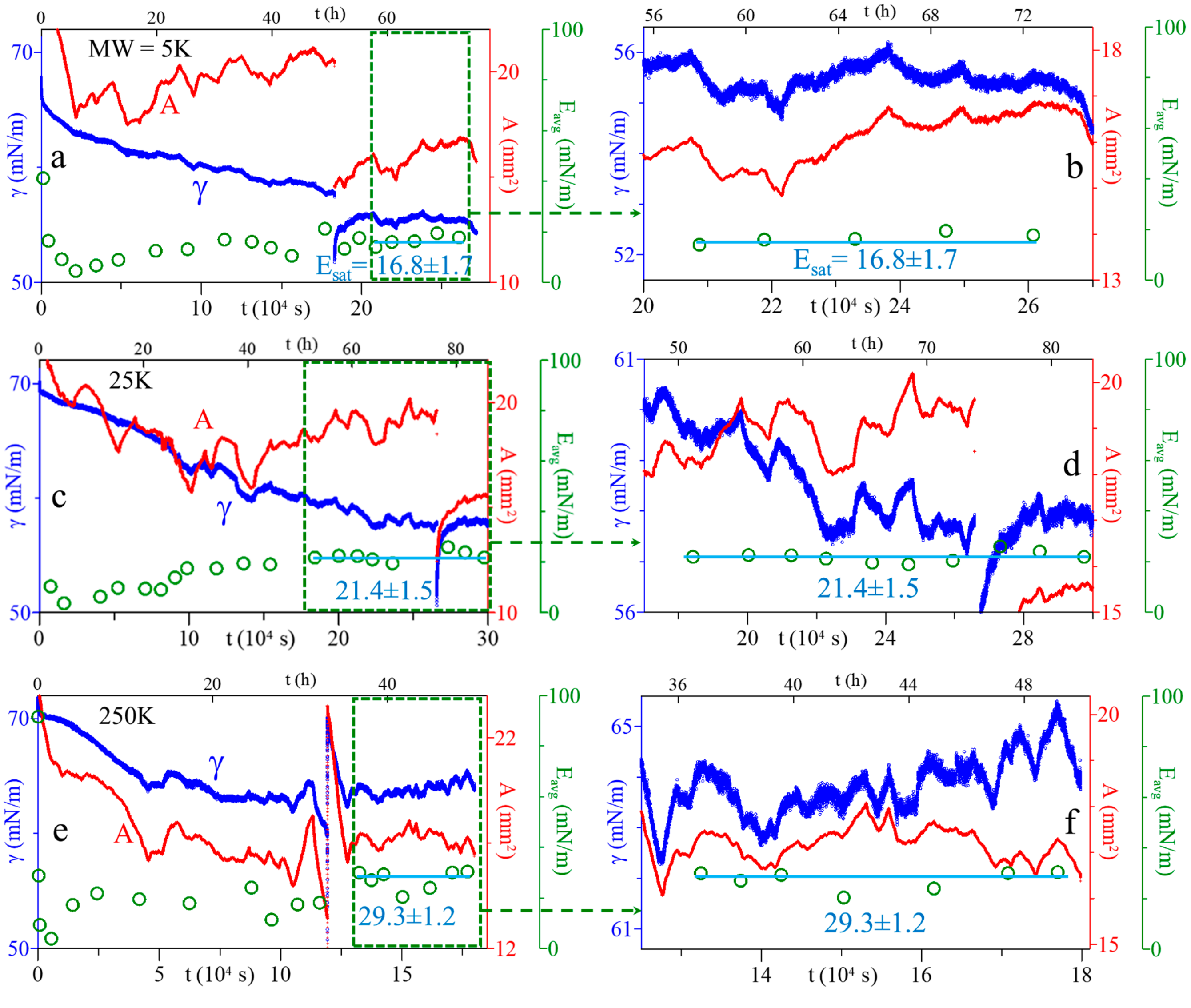

Figure S15. Variation of E_{avg} of PAA solutions alongside the corresponding relaxations of ST and bubble SA at C_{PAA} = 5×10^{-4} g/cm^3, MW = 5 (a-b), 25 (c-d), 250 (e-f) (kDa). The horizontal lines denote the E_{sat}.

S7. Comparison: E/E_{sat} of polymer films

A comparison between the E/E_{sat} of PAA+additive films reported in the literature and in this study was conducted (shown in Figure S16). Similar values, 10–40 mN/m, were obtained for the E or E_{sat} of PAA+additive films (at low additive concentration) (Figure 6 in manuscript). However, E was found to increase at increasing salt or surfactant additive (concentration or chain length) [1,8,10-12] (listed in Table S4, Figure S16). The E of PPG-r-PEG films was also reported to increase with polymer MW, although at a lower polymer MW range [1]. In addition, the PMMA solution of MW 30 kDa shows a similar E, 25 mN/m [12].

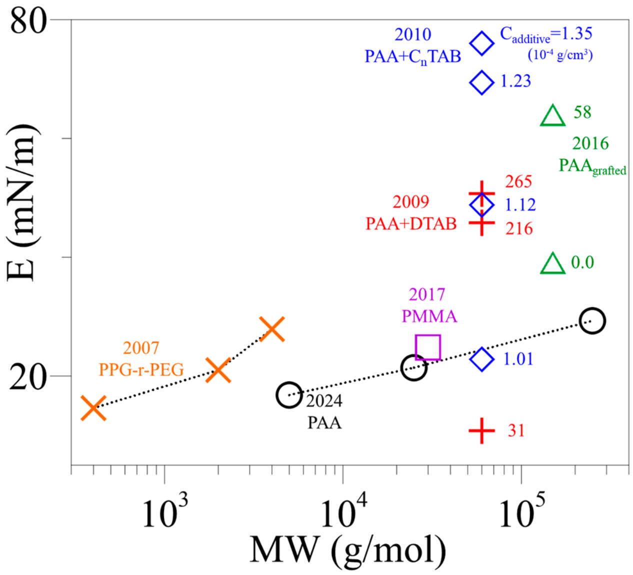

Figure S16. A comparison of the E/E_{sat} of polymer films reported in the literature and in this study.

Table S4. Reported E of polymer films compared with the E_{sat} of PAA films in this study (Figure S16).

Author, year	Compound	MW (kDa)	Conc. (10^{-4} g/cm^3)	Additive Conc.		E (mN/m)	Remarks
				10^{-4} g/cm^3	mol/cm^3		
Diez-Pascual, 2007	PPG-r-PEG	0.4	-	-	-	14.6	E_{max}
		2				21.0	
		4				27.9	
Bykov, 2009	PAA+DTAB	60	0.50	30.8	1.0×10^{-5}	10.8	E at t = 5 h
				216	7.0×10^{-5}	45.8	
				265	8.6×10^{-5}	50.7	
Bykov, 2010	PAA+C_8TAB	60	0.50	1.01	4.0×10^{-7}	22.8	E_{max}
	PAA+C_{10}TAB			1.12		48.8	
	PAA+C_{12}TAB			1.23		69.4	
	PAA+C_{14}TAB			1.35		76.0	
	PAA+C_{16}TAB			1.46		69.9	
Aricov, 2016	Grafted PAA + NaCl	150	7.50	-	-	39.1	pH=7.7
				292	5.0×10^{-4}	61.7	pH=5.9
				58.4	1.0×10^{-4}	64.0	pH=6.4
Gyurova, 2017	Modified PAA (with PMMA)	30	0.15	-	-	24.8	-
2024	PAA	5	5.00	-	-	16.8	E_{sat}
		25				21.4	
		250				29.3	

S8. Comparison: ST and E of aqueous PAA and globular protein solutions (BSA and HSA)

In the early stage (t < 6 h) of the PAA adsorption, E_{avg} was detected to decrease with time and reached a minimum of ~4 mN/m. To understand this behavior, the variation of E_{avg} of PAA films was compared with that of BSA (bovine serum albumin) and HSA (human serum albumin) films. This specific behavior, E_{avg} decreases in the early stage, reaches a minimum and then rises, can be detected for all PAA, BSA and HSA solutions (Figure S17). Moreover, many of the first E_{avg} were found to be larger than their respective E_{sat} (saturated films, listed in Table S5). This "significantly higher E_{avg} is likely not real but rather due to the significant contribution of [PAA/protein] adsorption (which caused a significant decrease in ST)" [14] at early adsorption.

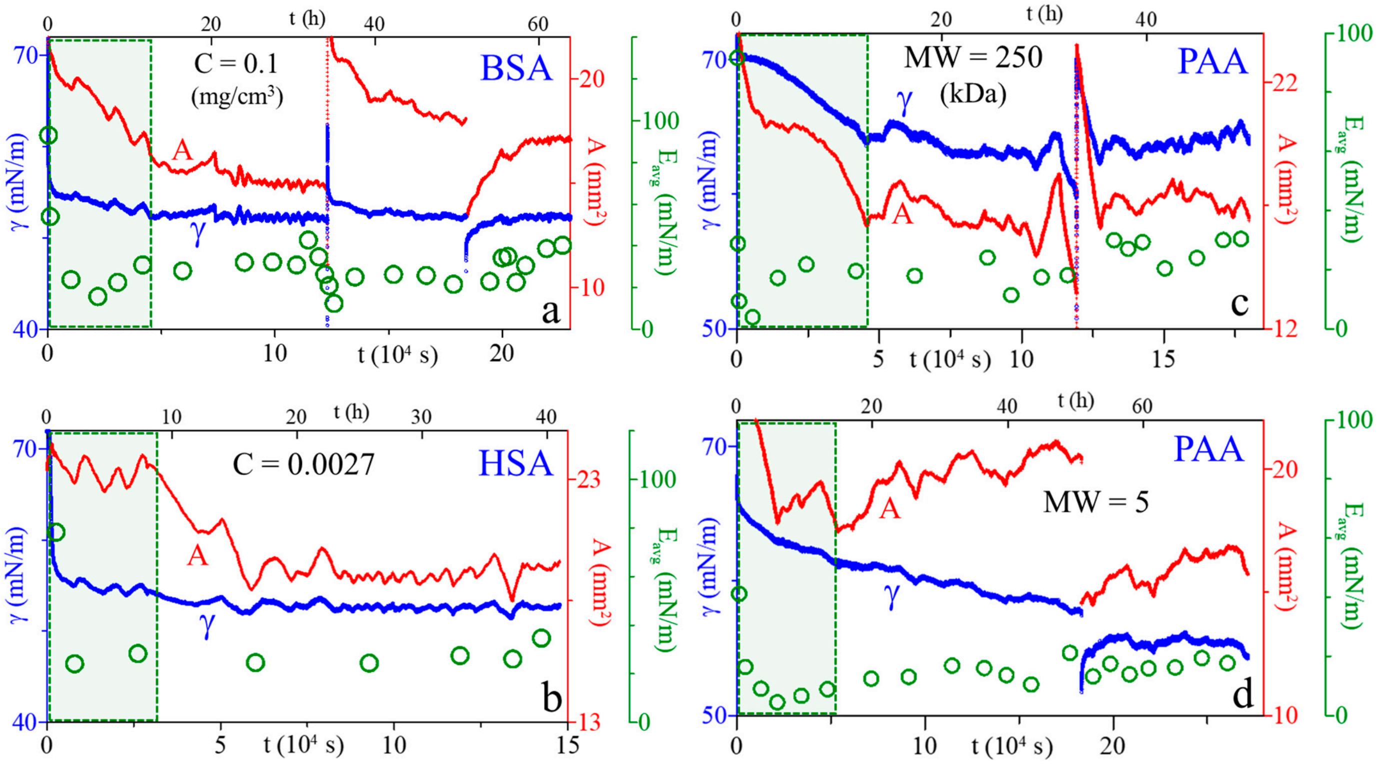

Figure S17. Variation in E_{avg} (circles) of PAA and protein solutions alongside their corresponding relaxations of ST and bubble SA at: (a) C_{BSA} = 0.1 (mg/cm^3); (b) C_{HSA} = 0.0027; (c) MW_{PAA} = 250 (kDa); and (d) MW_{PAA} = 5. The green boxes highlight the trend of E_{avg} observed in the early stage of adsorption.

The ST relaxations of BSA, HSA and PAA aqueous solutions are plotted together in Figure S18 for comparison. Note that the STs of BSA and HSA solutions relax much faster than those of PAA solutions (Figure S19, for solutions with nearly the same wt%).

Table S5. Comparison of MW, concentration, eq-ST and E_{sat} for different protein/polymer solutions.

Compound	MW (kDa)	Concentration		Eq-ST	E_{sat}
		(10^{-10} mol/cm^3)	(mg/cm^3)	(mN/m)	(mN/m)
BSA	66.4	0.052	0.00035	52.3	21.1
		15	0.10	52.3	40.7
		60	0.40	52.2	51.1
HSA	66.5	0.052	0.00035	52.6	22.1
		0.4	0.0027	52.7	34.7
		15	0.10	52.6	52.9
		60	0.40	52.7	50.4
PAA	5	1000	0.50	55.5	16.8
	25	200	0.50	57.9	21.4
	250	20	0.50	63.1	29.3

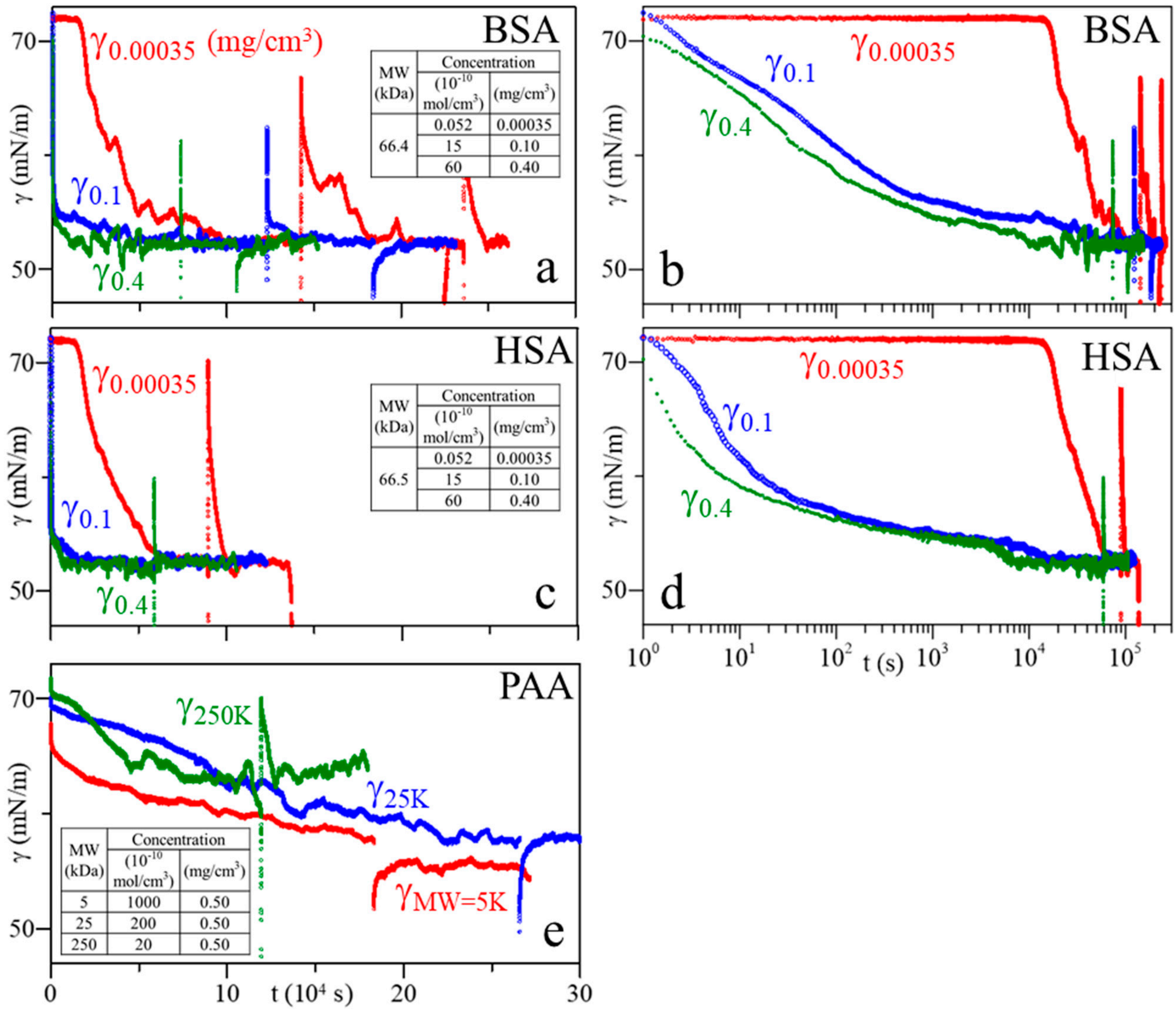

Figure S18. Surface tension (γ) relaxations of PAA and protein solutions at: (a-b) C_{BSA} = 0.00035, 0.1, 0.4 (mg/cm^3); (c-d) C_{HSA} = 0.00035, 0.1, 0.4; (e) MW_{PAA} = 5, 25, 250 (kDa).

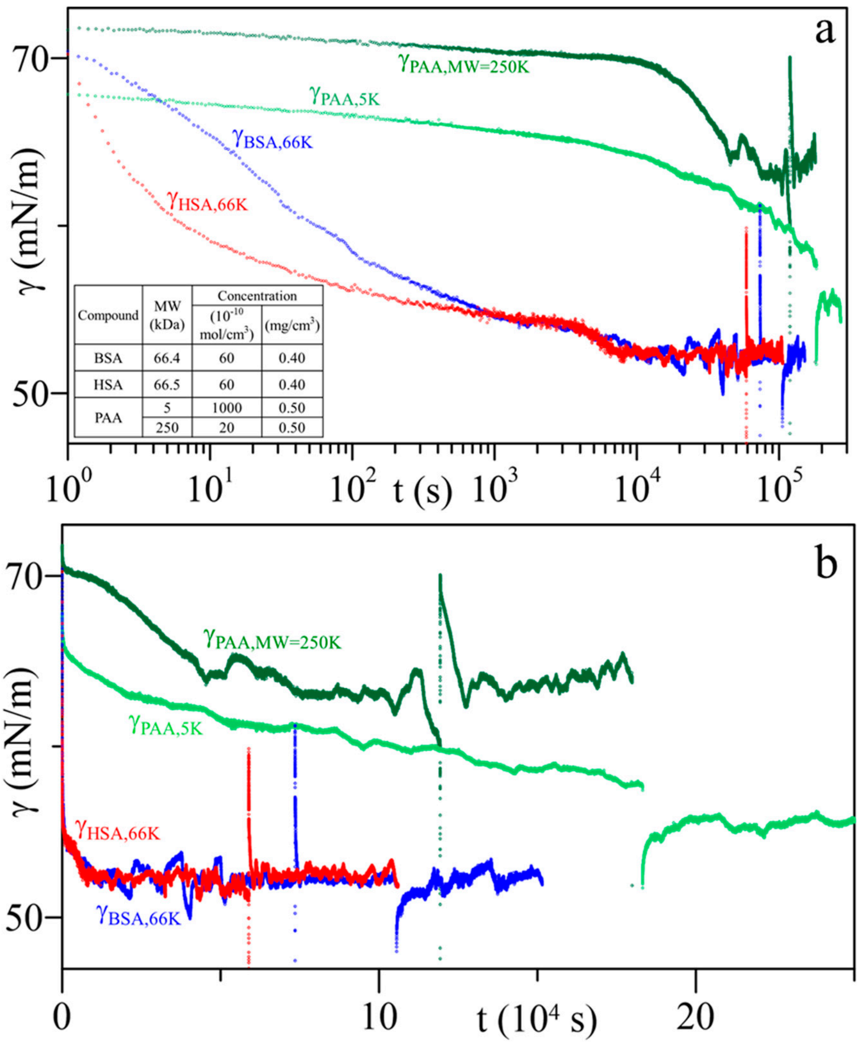

Figure S19. Surface tension (γ) relaxations of PAA and protein solutions of (a,b): MW_{PAA} = 5, 250 (kDa); MW_{BSA} = 66.4; and MW_{HSA} = 66.5.

The equilibrium-ST of aqueous PAA, BSA and HSA solutions and the E_{sat} of the adsorbed films were also compared (Figure S20). The γ_{eq} of PAA solutions is higher (55–63 mN/m) than that of BSA/HSA solutions (52–53 mN/m) (Figure S20a); whereas the adsorbed PAA film has a lower E_{sat} (17–30 mN/m) than that of globular protein films (~55 mN/m) (Figure S20b). Note that the E_{sat} for BSA and HSA solutions is a function of solution concentration (Table S5).

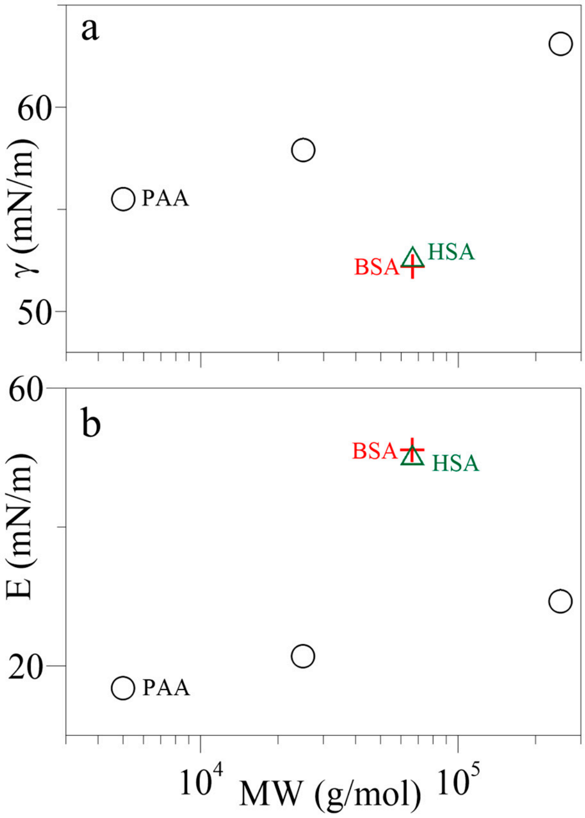

Figure S20. Variation of equilibrium-ST (a) of BSA/HSA/PAA solutions and the dilational modulus (E_{sat}) of their saturated adsorbed films (b) as a function of MW.

References

1. Diez-Pascual, A.; Monroy, F.; Ortega, F.; Rubio, R.; Miller, R.; Noskov, B. Adsorption of Water-Soluble Polymers with Surfactant Character. Dilational Viscoelasticity. *Langmuir* **2007**, *23*, 3802 – 3808.
2. Okumura, Y.; Kawaguchi, M. Surface pressure–area isotherms and surface dilational moduli of poly (N-isopropyl acrylamide) monolayers spread at air–water interface. *Colloids and Surfaces A: Physicochem. Eng. Aspects* **2014**, *441*, 275 – 280.
3. Noskov, B.A.; Bykov, A.G. Dilational surface rheology of polymer solutions. *Russian Chem. Reviews* **2015**, *84*, 634 – 652.
4. Noskov, B.A.; Nuzhnov, S.N.; Loglio, G.; Miller, R. Dynamic Surface Properties of Sodium Poly(styrenesulfonate) Solutions. *Macromolecules* **2004**, *37*, 2519 – 2526.
5. Milling, A.J.; Baines, F.L.; Armes, S.P.; Billingham, N.C.; Richards, R.W. Surface Viscoelastic Parameters of Poly((dimethylamino)ethyl methacrylate-methyl methacrylate) Diblock Copolymer Solutions: pH Dependence of the Evolution of the Equilibrium Values. *Macromolecules* **2001**, *34* 4173 – 4179.
6. Noskov, B. A.; Loglio, G.; Miller, R. Dilational Viscoelasticity of Polyelectolyte/Surfactant Adsorption Films at the Air/Water Interface: Dodecyltrimethylammonium Bromide and Sodium Poly(styrenesulfonate). *J. Phys. Chem., B* **2004**, *108*, 18615 – 18622.
7. Noskov, B.A.; Akentiev, A.V.; Bilibin, A.Yu.; Grigoriev, D.O.; Loglio, G.; Zorin, I.M.; Miller, R. Dynamic Surface Properties of Poly(N-isopropylacrylamide) Solutions. *Langmuir* **2004**, *20*, 9669 – 9676.
8. Bykov, A.G.; Lin, S.Y.; Loglio, G., Lyadinskaya, V.V.; Miller, R.; Noskov, B.A. Impact of surfactant chain length on dynamic surface properties of alkyltrimethylammonium bromide/polyacrylic acid solutions. *Colloids and Surfaces A: Physicochem. Eng. Aspects* **2010**, *354*, 382 – 389.
9. Lyadinskaya, V.V.; Bykov, A.G.; Campbell, R.A.; Varga, I.; Lin, S.Y.; Loglio, G.; Miller, R.; Noskov, B.A. Dynamic surface elasticity of mixed poly(diallyldimethylammonium chloride)/sodium dodecyl sulfate/NaCl solutions. *Colloids and Surfaces A: Physicochem. Eng. Aspects* **2014**, *460*, 3 – 10.
10. Bykov, A.G.; Lin, S.Y.; Loglio, G.; Miller, R.; Noskov, B.A. Kinetics of Adsorption Layer Formation in Solutions of Polyacid/Surfactant Complexes. *J. Phys. Chem. C* **2009**, *113*, 5664 – 5671.
11. Aricov, L.; Petkova, H.; Arabadzhieva, D.; Iovescu, A.; Mileva, E.; Khristov, K.; Stinga, G.; Mihailescu, C.F.; Anghel, D.F.; Todorov, R. Aqueous solutions of associative poly(acrylates): Bulk and interfacial properties. *Colloids and Surfaces A: Physicochem. Eng. Aspects* **2016**, *505*, 138 – 149.
12. Gyurova, A.Y.; Halacheva, S.; Mileva, E. Aqueous solutions of random poly(methyl methacrylate-co-acrylic acid): effect of the acrylic acid content. *RSC Advances* **2017**, *7*, 13372 – 13382.

13. Tseng, W.C.; Tsay, R.Y.; Le, T.T.Y.; Hussain, S.; Noskov, B.A.; Akentiev, A.; Yeh, H.H.; Lin, S.Y. Evaluation of the dilational modulus of protein films by pendant bubble tensiometry *J. Mol. Liq.* **2022**, *349*, 118113.
14. Hussain, S.; Rivas, J.E.M.; Tseng, W.C.; Tsay, R.Y.; Noskov, B.; Loglio, G.; Lin, S.Y. Measurement of Dilational Modulus of an Adsorbed BSA Film Using Pendant Bubble Tensiometry: From a Clean Interface to Saturation. *Coll. Interf.* **2024**, *8*, 4.
